# PIK3CA mutation status, progression and survival in advanced HR + /HER2- breast cancer: a meta-analysis of published clinical trials

**DOI:** 10.1186/s12885-022-10078-5

**Published:** 2022-09-21

**Authors:** Mirko Fillbrunn, James Signorovitch, Fabrice André, Iris Wang, Ines Lorenzo, Antonia Ridolfi, Jinhee Park, Akanksha Dua, Hope S. Rugo

**Affiliations:** 1 Analysis Group, Inc., Boston, Massachusetts USA; 2 Institut Gustave Roussy, Villejuif, France; 3 Novartis, East Hanover, New Jersey, USA; 4 Novartis, Madrid, Spain; 5 Novartis, Paris, France; 6 University of California San Francisco Helen Diller Family Comprehensive Cancer Center, San Francisco, California USA

**Keywords:** Hormone receptor positive/human epidermal receptor 2 negative (HR + /HER2-), Metastatic breast cancer (mBC), *PIK3CA*, Overall survival, Progression-free survival

## Abstract

**Background:**

Approximately 40% of hormone receptor positive/human epidermal receptor 2 negative (HR + /HER2-) metastatic breast cancer (mBC) patients harbor phosphatidylinositol-4,5-bisphosphate 3-kinase catalytic subunit alpha (*PIK3CA*) mutations. However, associations between *PIK3CA* mutation status and clinical outcomes among patients with HR + /HER2- mBC have been heterogeneous across clinical trials. This meta-analysis was conducted to survey recently available trial data to assess the prognostic effects of *PIK3CA* among patients with HR + /HER2- mBC.

**Methods:**

Randomized clinical trials reporting progression-free survival (PFS) or overall survival (OS) stratified by *PIK3CA* status in HR + /HER2- mBC were identified via systematic literature review. Trial arms receiving phosphatidylinositol 3-kinase (*PI3K)-*targeted therapies were excluded. Meta-regression analysis was used to estimate the association between PIK3CA status and PFS and OS among included studies.

**Results:**

The analyzed data included 3,219 patients from 33 study arms across 11 trials (*PIK3CA* mutated: 1,386, wild type: 1,833). *PIK3CA* mutation was associated with shorter median PFS (difference [95% CI] (months): -1.8 [-3.4, -0.1], I^2^ = 35%) and shorter median OS (-8.4 [-13.4, -3.5], I^2^ = 58%, *N* = 1,545). Findings were similar for PFS rates at 6 months (odds ratio [95% CI]: 0.74 [0.59, 0.94], I^2^ = 42%, *N* = 3,160) and 12 months (0.76 [0.59, 0.99], I^2^ = 42%, *N* = 2,468) and directionally consistent but not statistically significant at 18 months (*N* = 1,726).

**Conclusions:**

Pooling evidence across multiple studies, *PIK3CA* mutation was associated with shorter PFS and OS. These findings suggest a negative prognostic value of *PIK3CA* mutations in patients with HR + /HER2- mBC.

**Supplementary Information:**

The online version contains supplementary material available at 10.1186/s12885-022-10078-5.

## Background

Breast cancer (BC) is the most common form of cancer in women worldwide [1]. In the United States (US), a woman has a 1-in-8 chance of being diagnosed with BC in her lifetime, and in 2022 there were more than 280,000 new cases of BC [2]. Metastatic breast cancer (mBC), a BC that has spread beyond the breast and nearby lymph nodes, is generally incurable with a 5-year survival rate of 27% [2–5].

Treatment for mBC has been successively revolutionized by the targeting of molecular genetic factors, particularly tumors presenting as hormone receptor positive (HR +) or human epidermal growth factor receptor 2 positive (HER2 +). Approximately 60% of mBC cases are classified as HR + /HER2 negative (HER2-) [6].

Within this HR + /HER2- group, one of the most commonly mutated genes, with an estimated prevalence of 40%, is phosphatidylinositol-4,5-bisphosphate 3-kinase catalytic subunit alpha (PIK3CA), which encodes the p110α isoform of phosphatidylinositol 3-kinase (PI3K) [7, 8]. Abnormal signalling through the PI3K pathway relates to tumorigenesis, progression, and therapeutic resistance, which suggests prognostic relevance of PIK3CA mutations for patients with mBC [9–11]. Measuring the prognostic value of PIK3CA is central to assessing the clinical burden of this important subpopulation within HR + /HER2- mBC, and for contextualizing the outcomes of novel treatments targeting PIK3CA.

Several clinical trials have reported outcomes for patients with HR + /HER2- mBC classified by PIK3CA mutation status [12–14]. The apparent associations between mutation status and outcomes, however, have been heterogeneous. Differences in underlying patient populations and study design factors such as follow-up duration, mutation testing methodologies (circulating tumor DNA [ctDNA] or tissue testing), and background study treatments can complicate assessment of PIK3CA’s prognostic value across studies. There is a need to synthesize available evidence from these studies with attention to factors that might impact the relationship between PIK3CA mutations and outcomes [10, 15].

In the present study, we compared progression-free survival (PFS) and overall survival (OS) by PIK3CA status across studies conducted among patients with HR + /HER2- mBC, with adjustment for potential confounding or moderating effects of treatment type and mutation testing method. This study included only those trial arms that were free of PI3K-targeted treatments so that the prognostic effects of PIK3CA could be determined in the absence of such treatments.

## Methods

### Systematic literature review process

**Fig. 1:**
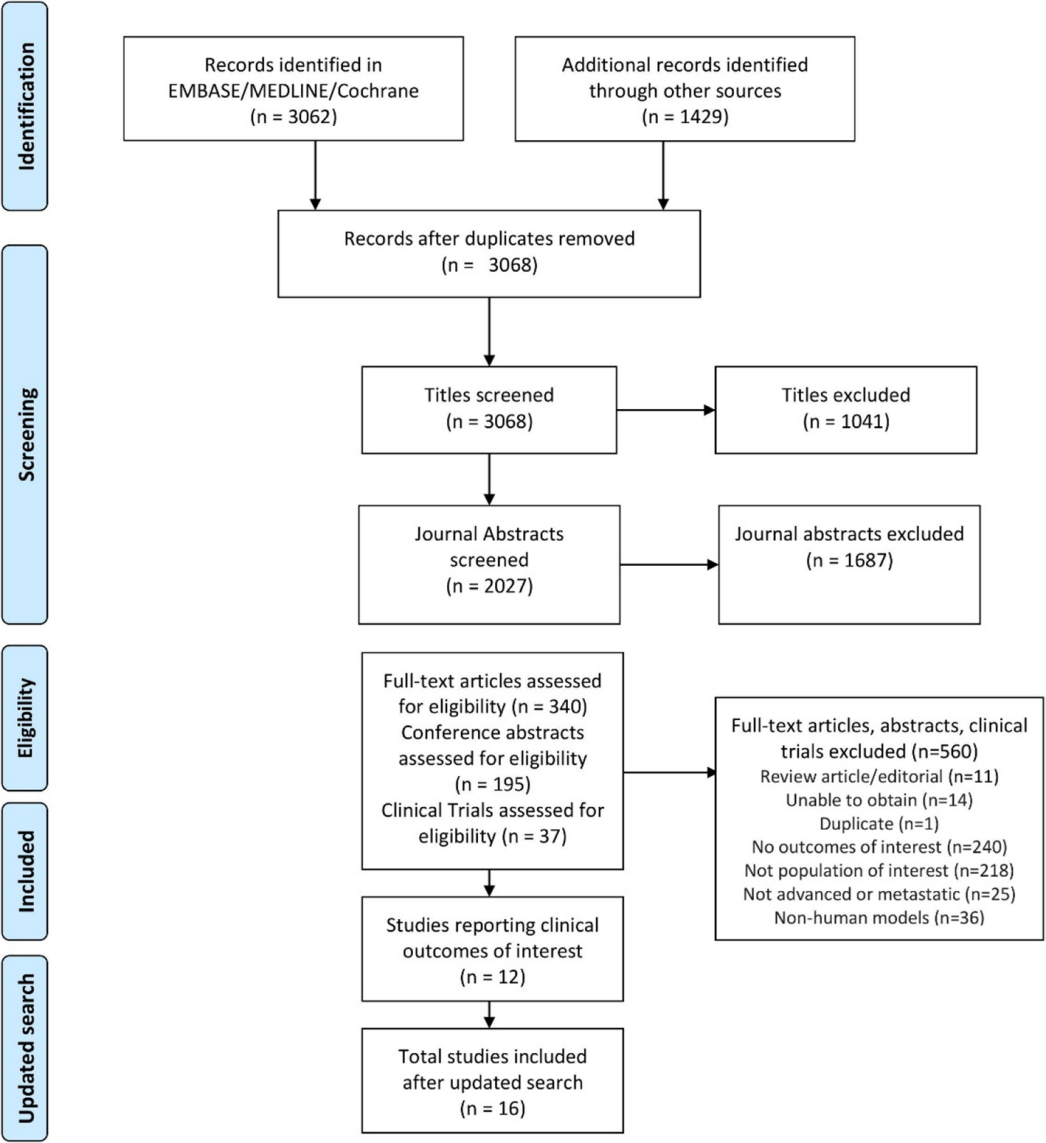
Preferred Reporting Items for Systematic Reviews and Meta-Analysis guidelines diagram for electronic search

A systematic literature review was conducted by searching the MEDLINE and MEDLINE In-Process, EMBASE, Cochrane databases, and the Database for Abstracts of Reviews of Effects, for articles published from January 1993 to April 2019 [16]. Additional manual searches to complement the electronic search were conducted until April 2022. All databases were searched through the Ovid platform. The full search strategy and screening criteria for the electronic search can be found in sections 1 and 2 of the Supplementary Material. Preferred Reporting Items for Systematic Reviews and Meta-Analysis guidelines were followed in designing, performing, and reporting the systematic review (Fig. 1). Abstract books from key congresses, bibliographies from previous systematic literature reviews, and ClinicalTrials.gov were hand-searched to supplement the electronic search. Screening for inclusion was conducted by two reviewers working independently.

### Inclusion criteria

Studies were eligible for inclusion if they were conducted in human subjects or with human tissue, included post-menopausal women with HR + /HER2- mBC, contained information on the presence of PIK3CA mutation, included patients treated with monotherapy or combination therapy, reported PIK3CA mutation status among the HR + /HER2- subgroup, and reported results on PFS or OS stratified by PIK3CA mutation status.

### Exclusion criteria

As the objective of this meta-analysis was to identify the prognostic value of PIK3CA mutation in the absence of PI3K-targeted therapies, trial arms that included PI3K-targeted therapies (alpelisib, buparlisib, taselisib, and pictilisib) were excluded from the analysis. Data from non-PI3K -targeted comparator arms remained included. Additionally, research in other disease areas has shown differences in outcomes between patients with amplifications and mutations, so studies that reported only combined statistics on PIK3CA mutations and amplifications were excluded from the analysis [17].

### Feasibility assessment and data extraction

Publications identified in the systematic literature review were assessed for their suitability in terms of data availability and clinical relevance for inclusion in the meta-analysis. Summaries of trial characteristics (e.g., sample size, study design, follow-up, key inclusion and exclusion criteria, prior therapies), baseline characteristics (e.g., demographic characteristics [age, sex, race], clinical characteristics [menopausal status, Eastern Cooperative Oncology Group score, co-mutations], and PIK3CA testing methodologies), and outcome measures (PFS, OS, and Kaplan–Meier [KM] survival curves) were independently extracted by two reviewers. Any discrepancies in the extraction were evaluated by a third reviewer and resolved through discussion until consensus was reached. A complete list of included studies and corresponding data availability is presented in Table 1 and Table S1 in the Supplemental Material.

**Table 1:** Publications included in data extraction

Publication title	First Author, year	Study acronym
Alpelisib plus fulvestrant for PIK3CA-mutated, hormone receptor-positive, human epidermal growth factor receptor-2enegative advanced breast cancer: final overall survival results from SOLAR-1	Andre, 2021 [18]	SOLAR-1
Alpelisib (ALP) with fulvestrant (FUL) in patients (pts) with PIK3CA-mutated hormone receptor-positive (HR +), human epidermal growth factor receptor-2-negative (HER2-) advanced breast cancer (ABC): Primary or secondary resistance to prior endocrine therapy (ET) in the SOLAR-1 trial	Juric, 2019 [19]	SOLAR-1
Buparlisib plus fulvestrant versus placebo plus fulvestrant in postmenopausal, hormone receptor-positive, HER2-negative, advanced breast cancer (BELLE-2): a randomised, double-blind, placebo-controlled, phase 3 trial	Baselga, 2017[15]	BELLE-2
Buparlisib plus fulvestrant versus placebo plus fulvestrant for postmenopausal, hormone receptor-positive, human epidermal growth factor receptor 2-negative, advanced breast cancer: Overall survival results from BELLE-2	Campone, 2018 [20]	BELLE-2
Fulvestrant plus palbociclib versus fulvestrant plus placebo for treatment of hormone-receptor-positive, HER2-negative metastatic breast cancer that progressed on previous endocrine therapy (PALOMA-3): final analysis of the multicentre, double-blind, phase 3 randomised controlled trial	Cristofanilli, 2016[21]	PALOMA-3
Overall Survival with Palbociclib and Fulvestrant in Advanced Breast Cancer	Turner, 2018 [22]	PALOMA-3
Buparlisib plus fulvestrant in postmenopausal women with hormone-receptor-positive, HER2-negative, advanced breast cancer progressing on or after mTOR inhibition (BELLE-3): a randomised, double-blind, placebo-controlled, phase 3 trial	Di Leo, 2018 [23]	BELLE-3
Phase II Study of Taselisib (GDC-0032) in Combination with Fulvestrant in Patients with HER2-Negative, Hormone Receptor–Positive Advanced Breast Cancer	Dickler, 2018 [24]	
Phase II trial of temsirolimus in patients with metastatic breast cancer	Fleming, 2012 [25]	
Correlative Analysis of Genetic Alterations and Everolimus Benefit in Hormone Receptor–Positive, Human Epidermal Growth Factor Receptor 2–Negative Advanced Breast Cancer: Results From BOLERO-2	Hortobagyi, 2016 [26]	BOLERO-2
Pictilisib for oestrogen receptor-positive, aromatase inhibitor-resistant, advanced or metastatic breast cancer (FERGI): a randomised, double-blind, placebo-controlled, phase 2 trial	Krop, 2016 [27]	FERGI
Stand Up to Cancer Phase Ib Study of Pan-Phosphoinositide3-Kinase Inhibitor Buparlisib With Letrozole in Estrogen Receptor-Positive/Human Epidermal Growth Factor Receptor 2-Negative Metastatic Breast Cancer	Mayer, 2014 [28]	
A Phase Ib Study of Alpelisib (BYL719), a PI3Kα-specific Inhibitor, with Letrozole in ER + /HER2-Negative Metastatic Breast Cancer	Mayer, 2017 [29]	
Correlation between PIK3CA mutations in cell-free DNA and everolimus efficacy in HR + , HER2- advanced breast cancer: results from BOLERO-2	Moynahan, 2017 [30]	BOLERO-2
Natural history and outcome of patients presenting a metastatic breast cancer (mBC) with PIK3CA mutation	Mosele, 2019 [31]	SAFIR02_Breast
Phase III study of Taselisib (GDC-0032) + fulvestrant (FULV) v FULV in patients with ER + , PIK3CA-mutant, locally advanced or metastatic breast cancer: Primary analysis from SANDPIPER	Baselga, 2018 [13]	SANDPIPER
Clinical Significance of PIK3CA and ESR1 Mutations in Circulating Tumor DNA: Analysis from the MONARCH 2 Study of Abemaciclib plus Fulvestrant	Tolaney, 2022 [32]	MONARCH 2

This study only used previously published summary statistics from the included studies. The risk of bias of individual studies was assessed elsewhere, and all studies were found to be of low to moderate risk [16]. No institutional review board approval was required.

### Outcome measures

The outcomes of interest in this study were PFS, defined as the time from randomization until objective disease progression (all included trials used RECIST v1.0 or v1.1) or death, and OS, defined as the time from randomization until death from any cause. PFS and OS were extracted as the median time to event (point estimates and associated 95% confidence intervals [CI; months]) and as KM curves when available. For PFS, 6-, 12-, and 18-month rates were extracted from KM curves. In particular, time-to-event outcomes were re-constructed from published KM curves using a standard digitization approach (see section 3 of the Supplementary Material for technical details) [33, 34]. Not all study arms that reported median OS reported an associated KM curve, in which case standard errors for median OS were approximated using the reported 95% CIs given the asymptotic normal distribution [35]. Comparing the two methods (imputation via KM curves and via CIs) on study arms where both were available, the standard errors using CIs were on average 12% larger than those estimated from extracted KM curves, which thus did not lead to an overestimation of precision for studies with missing KM curves.

### Analysis variables

Binary variables (e.g., PIK3CA mutation status) were extracted as frequencies and percentages at the study level, whereas continuous variables (e.g., follow-up time) were extracted as means or medians with standard errors where available.

### Statistical analysis

#### Meta-analyses

A meta-regression analysis was used to estimate the association between PIK3CA mutation status and PFS (median PFS and 6-, 12-, and 18-month PFS rates) or median OS across included studies (see Sect. 5 of the Supplemental Materials Table S2-S9 for detailed results). A multi-level mixed-effects model was used, with study-level random intercepts and random coefficients on PIK3CA status to capture the heterogeneity in effect sizes due to differences not explained by meta-regression factors [36]. In this meta-analysis, multiple analyses of the same or overlapping trial population could be included. For example, two clinical trials (SOLAR-1 and BOLERO-2) had multiple publications included. However, the data from these publications are not duplicates of one another as they differed in which testing method was used to determine PIK3CA mutation status. These differences in testing method can lead to different assignments of PIK3CA mutation and wild-type cohorts between the publications and therefore different inputs into our meta-analysis and provide distinct yet correlated information [15]. Trial-level random effects were included to account for correlation arising from the analyses of such overlapping analyses [37]. Due to the inclusion of multiple studies of the same trial, the number of unique patients may be lower than the sum across all studies.

The meta-regression model employed inverse-variance weighting and was estimated using restricted maximum likelihood (REML) using R (version 3.6.1). Test statistics and CIs were based on *t*-distributions, and the magnitude of differences between mutated and wild-type cohorts were interpreted via Cohen’s d effect size using the pooled standard deviation of all studies [38]. As is common, differences of size 0.2 were considered small, 0.5 represented moderate differences, and 0.8 represented large differences. Heterogeneity was assessed via Cochran’s Q statistic and between-cluster I^2^, which estimates the ratio of the observed variability in effect sizes across studies that is due to heterogeneity in true effect sizes (i.e., study- or trial-level effects) rather than sampling variance [39].

#### Model specifications

PIK3CA mutation status was studied as the primary exposure variable in the meta-regression model. Median PFS and OS times were studied on a linear scale and PFS rates at 6-, 12-, and 18-months were studied on a logit scale. These models, without further adjustment for arm- or trial-level factors, were studied as the primary analysis for each outcome. For OS data, limited data availability prevented further analyses. For PFS data, meta-regression adjustments were used to explore potential confounders or modifiers of the relationship between PIK3CA status and PFS outcomes: testing methodology used to determine PIK3CA status (tissue testing vs. ctDNA testing) and study arm treatment (placebo + fulvestrant (ful), palbociclib + ful, everolimus + exemestane, abemaciclib + ful, other [targeted therapy, standard maintenance chemotherapy, or immunotherapy]). Analyses adjusting for treatment categorized as including vs. not including fulvestrant were also conducted. Effect modifiers were studied by adding them one at a time into the meta-regression model, first as a main effect and then as a main effect including an interaction term with PIK3CA mutation status.

## Results

### Included studies and baseline characteristics

Of 3062 identified articles, conference abstracts, and posters, 572 full-text articles were reviewed. An updated targeted literature search through April 2022 identified two new publications with updated data (later data cut-offs) for included trials (SOLAR-1 and MONARCH-2). A total of 33 study arms across 11 unique trials (17 publications) were selected and included in the meta-analysis. A total of 3,219 patients were included across the trials, with 1,386 patients in the PIK3CA-mutated cohort and 1,833 patients in the wild-type cohort. Sample sizes may differ based on analysis specifications. Study designs and patient characteristics are described in Table 2.

**Table 2:** Aggregated trial characteristics^a,b^

		**Study cohorts, stratified by PIK3CA mutation status**	
	**Full Sample**	**Mutated**	**Wild**
	***N* = 33**	***N* = 17**	***N* = 16**
**Study and Patient characteristics**			
Total number of patients	3,219	1,386	1,833
**PIK3CA Mutation Testing Methodology, N (%)**			
ctDNA testing	18 (54.5%)	9 (52.9%)	9 (56.3%)
Tissue testing	13 (39.4%)	7 (41.2%)	6 (37.5%)
Missing	2 (6.1%)	1 (5.9%)	1 (6.3%)
**Percentage of Patients with Prior Chemotherapy, median (range)**			
Overall^c^	67.0 (27.4, 95.4)	64.6 (27.4, 92.0)	72.0 (29.8, 95.4)
For metastatic disease^c^	27.8 (0.0, 35.0)	27.4 (0.0, 34.0)	29.9 (0.0, 35.0)
**Median Follow-up Time (Months)**			
Overall	14.6 (7.4, 45.1)	14.6 (8.9, 45.1)	14.5 (7.4, 45.1)
**Study treatment**			
Any Fulvestrant	23 (69.7%)	12 (70.6%)	11 (68.8%)
Abemaciclib + Fulvestrant	2 (6.1%)	1 (5.9%)	1 (6.3%)
Placebo + Fulvestrant	19 (57.6%)	10 (58.8%)	9 (56.3%)
Palbociclib + Fulvestrant	2 (6.1%)	1 (5.9%)	1 (6.3%)
Everolimus + Exemestane	4 (12.1%)	2 (11.8%)	2 (12.5%)
Placebo + Exemestane	4 (12.1%)	2 (11.8%)	2 (12.5%)
Other	2 (6.1%)	1 (5.9%)	1 (6.3%)
**Survival outcomes**			
Median progression-free survival (months)	5.6 (1.4, 23.4)	5.4 (1.4, 19.0)	6.2 (1.7, 23.4)
6 Month Survival Rate (%)^e^	46.2 (10.8, 93.4)	43.0 (10.8, 91.0)	53.5 (19.8, 93.4)
12 Month Survival Rate (%)^e^	31.7 (5.8, 76.4)	29.2 (18.8, 66.7)	32.6 (5.8, 76.4)
18 Month Survival Rate (%)^e^	22.7 (3.2, 64.8)	20.0 (18.4, 51.3)	26.4 (3.2, 64.8)
Median overall survival (months)^d^	32.2 (19.6, 55.5)	26.9 (19.6, 44.5)	37.8 (23.5, 55.5)

*Abbreviations*: *ctDNA*, circulating tumor deoxyribose nucleic acid, *PIK3CA* Phosphatidylinositol-4,5-bisphosphate 3-kinase catalytic subunit alpha

*Notes:* ^a^Medians and ranges are shown for continuous characteristics, counts and percentages are shown for categorical characteristics. Percentages may not total 100 because of rounding

^b^Unit of observation is study cohort, which refers to subpopulation of study arms based on PIK3CA mutation status

^c^Medians and ranges calculated from available data. Overall percentages of patients with prior chemotherapy were available for 11 study cohorts (mutated (MT): 6, wild (WT): 5), percentages for metastatic disease were available for 9 study cohorts (MT: 5, WT: 4)

^d^Medians and ranges calculated from available data. Median OS were available for 14 study cohorts (MT: 8, WT: 6)

^e^Medians and ranges calculated from available data. PFS rates at 6 months were available for 31 study cohorts (MT: 16, WT: 15), at 12 months for 22 study cohorts (MT: 10, WT: 12), at 18 months for 14 study cohorts (MT: 7, WT: 7)

Median follow-up time was 14.6 months (7.4, 45.1) and was comparable across the two cohorts (differing by less than 1 month). PIK3CA mutation status was determined by ctDNA testing in 18 study cohorts (54.5%) and via tissue testing in 13 (39.4%), while for 2 study cohorts (6.1%) both methods were used without further information on the number of patients tested with each method. Most study cohorts (*N* = 23 [69.7%]) included fulvestrant as part of their treatment regimen; most of these fulvestrant arms were trial comparator arms and included a placebo plus fulvestrant (19 [57.6%]), while the remaining four fulvestrant arms included abemaciclib (2 [6.1%]) or palbociclib (2 [6.1%]). Other treatments included exemestane with everolimus (4 [12.1%]) or with placebo (4 [12.1%]) or targeted therapy, standard maintenance chemotherapy with or without immunotherapy (2 [6.1%]).

### Median PFS

The analyzed data included 3219 patients from 33 study arms (PIK3CA-mutated: 1386, wild-type: 1833). Across all included studies, the median PFS as reported by the studies had a median of 5.6 months (range: 1.4, 23.4) for the overall cohort, with a 5.4 months (1.4, 19.0) for the PIK3CA-mutated cohort compared to 6.2 months (1.7, 23.4) for the PIK3CA wild-type cohort. PFS rates at 6, 12 and 18 months were similar in the PIK3CA-mutated and wild-type cohorts and had medians of 46.2% (10.8%, 93.4%), 31.7% (5.8%, 76.4%), and 22.7% (3.2%, 64.8%), respectively.

**Fig. 2:**
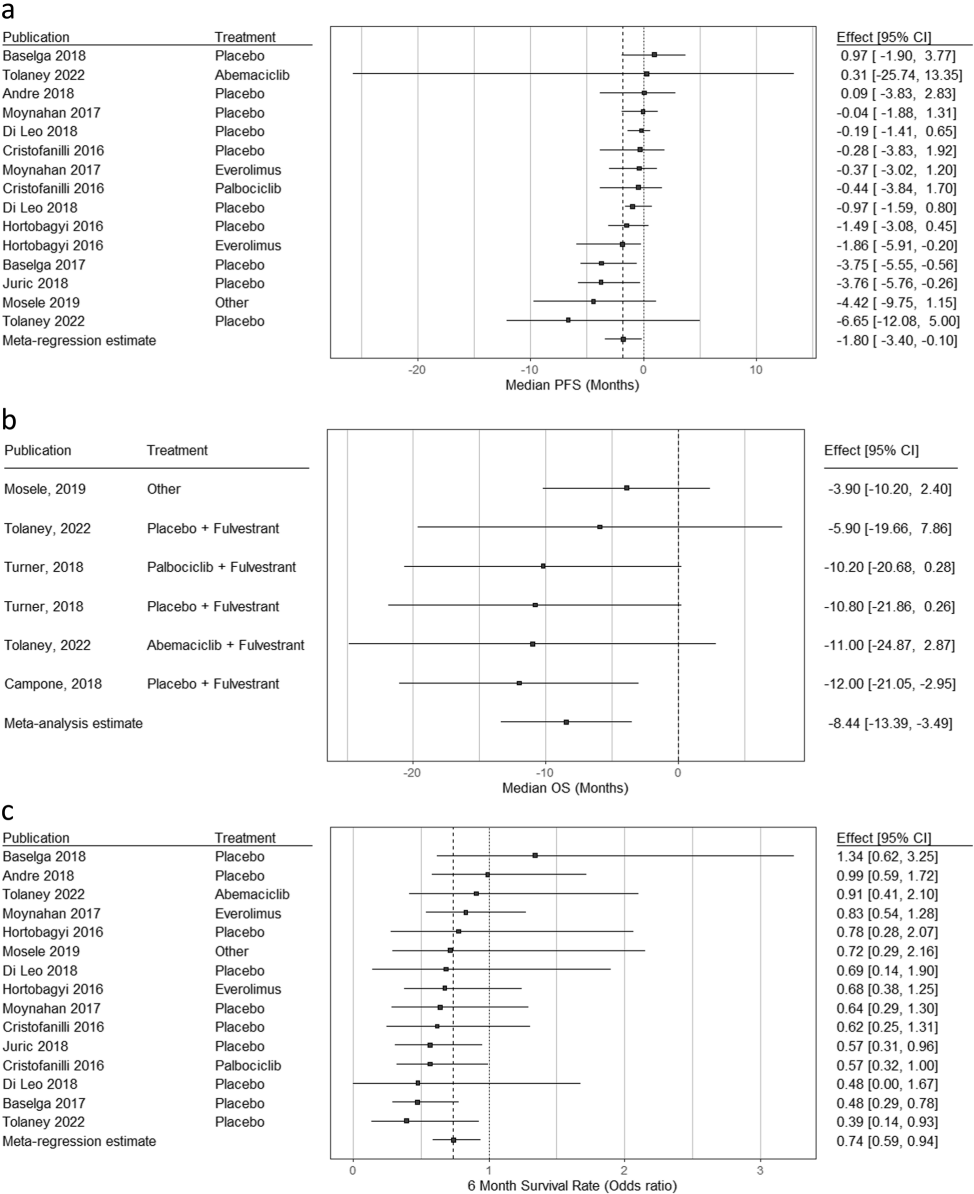
Within-study differences between PIK3CA-mutated and wild-type cohorts (**a**) PFS medians (**b**) OS medians (**c**) 6-month PFS rate (odds ratio). Abbreviations: CI, confidence interval; PFS, progression-free survival; PIK3CA, phosphatidylinositol-4, 5-bisphosphate 3-kinase catalytic subunit alpha; OS, overall survival

Differences in median PFS between the PIK3CA-mutated and wild-type cohorts within studies are represented in Fig. 2a. For all studies except Baselga et al. 2018 [13], Andre et al. 2018 [40], and Tolaney, 2022 [32], patients in the PIK3CA wild-type cohort had a longer median PFS compared to patients in the PIK3CA-mutated cohort. The within-study differences in the median PFS between the PIK3CA-mutated and wild-type cohorts ranged from 0.97 months to -6.65 months.

**Fig. 3:**
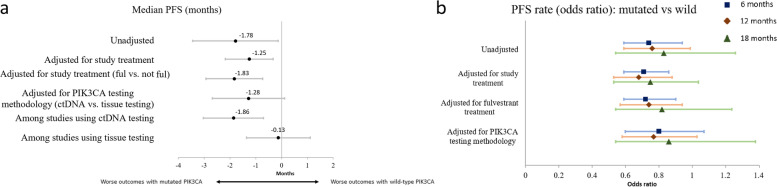
Associations between PIK3CA status and PFS outcomes. **a** Median PFS. **b** PFS rates. Abbreviations: ctDNA, circulating tumor deoxyribonucleic acid; PFS, progression-free survival; PIK3CA, phosphatidylinositol-4,5-bisphosphate 3-kinase catalytic subunit alpha

In the meta-regression model combing data across studies, mutated PIK3CA was associated with shorter median PFS (difference [95% CI]: -1.8 [-3.4, -0.1] months, Cohen’s d = 0.3). Cross-study differences accounted for a minority of the variability in median PFS differences (*I*^*2*^ = 34.5%). The direction of this association was robust to adjustment for study treatment (Fig. 3a). When adjusting for testing methodology (tissue testing vs. ctDNA testing) and dropping two study arms without information on testing methodology, mutated PIK3CA status was numerically associated with a lower median PFS (-1.3 [-2.7, 0.1], d = 0.3); this association was not statistically significant. However, the model with an interaction between PIK3CA status and testing method identified significant modification of the PIK3CA-PFS association by testing method (*P* < 0.05). The association was stronger in the subgroup of studies using ctDNA testing (-1.9 [-3.0, -0.7], d = 0.5, total patients N: 1857) than in the subgroup of studies using tissue-testing (-0.1 [-1.4, 1.1], d = 0.03, N: 998).

### Median OS

The analysis of median OS included 1545 patients from 14 study arms (PIK3CA-mutated: 708, wild-type: 837). Median OS was 32.2 months (range: 19.6, 55.5), with a 26.9 months (19.6, 44.5) for the eight PIK3CA-mutated cohorts compared to 37.8 months (23.5, 55.5) for the six PIK3CA wild-type cohorts. Median follow-up time across study arms with OS was 30.8 months (*N* = 2 study arms did not report follow-up).

Figure 2b presents differences in median OS between the PIK3CA-mutated and wild-type cohorts within studies. In all studies, patients in the PIK3CA wild-type cohort had a longer median OS compared to patients in the PIK3CA-mutated cohort, ranging from a difference of 3.9 months to 12.0 months. In the unadjusted meta-regression model, mutated PIK3CA was associated with shorter median OS (-8.4 [-13.4, -3.5], d = 0.9) and cross-study differences accounted for *I*^*2*^ = 58.2% of the variability in median OS differences.

### PFS rates

PFS rates at 6 months (see Fig. 2c), 12 months, and 18 months were analyzed (see Fi.g S1). The 6-month data included 3160 patients from 31 study arms across 11 publications (PIK3CA-mutated: 1366; PIK3CA wild-type: 1794). Fewer studies reported rates at 12 months (N: 2468; mutated: 1056, wild-type: 1412) and 18 months (N: 1726; mutated: 811, wild-type: 915). Differences in PFS rates between the PIK3CA-mutated and wild-type cohorts within studies are represented in Fig. 2c and Fig. S1 in the Supplementary Material. For most studies, patients in the PIK3CA wild-type cohort had higher PFS rates compared to patients in the PIK3CA-mutated cohort at 6, 12, and 18 months. The odds ratios of 6-month PFS rates between the mutated and wild-type cohorts ranged from 0.39 to 1.34 across the 15 study arms for which both mutated and wild-type KM curves were available, for 12 months between 0.47 to 3.08 across 8 study arms, and for 18 months between 0.45 to 1.06 across 5 study arms.

In the meta-regression models for the association between PIK3CA mutation status and logit-transformed PFS rates, mutated PIK3CA status was overall associated with lower odds of PFS compared to PIK3CA wild-type at 6 months (odds ratio [95% CI]: 0.74 [0.59, 0.94], *I*^*2*^ = 41.7%) and at 12 months (0.76 [0.59, 0.99], *I*^*2*^ = 42.0%), and directionally consistent but not statistically significant at 18 months (0.83 [0.54, 1.26], *I*^*2*^ = 31.7%). This association was robust to adjustment for study treatment at 6 and 12 months. When adjusting for testing methodology (tissue testing vs. ctDNA testing), the association was directionally consistent but not statistically significant for 6 months (0.80 [0.60, 1.07]) or 12 months (0.77 [0.58, 1.03]) (Fig. 3b). Models including interactions between testing methodology or treatment and mutation status did not identify significant effect modification.

## Discussion

Patients with mBC have a known poor prognosis, with a life-expectancy of approximately 3 years, as well as a high cost burden of treatment and annual estimated treatment costs of almost $60,000 per patient in the United States [41]. In addition to the significant clinical and economic burden incurred by patients with mBC, progression of metastatic disease is further associated with poor workplace productivity, lower probability of employment, and increased workplace hours missed [42]. This study systematically reviewed and synthesized evidence on prognostic associations between PIK3CA mutation status and PFS and OS across clinical trials in HR + /HER2- mBC. While the included studies had differences in patient populations, mBC treatments, and PIK3CA testing methods, we identified evidence of shorter PFS and OS times among patients with mutated PIK3CA compared to wild-type. In addition, among the studies reporting median PFS and testing type, we identified evidence that the testing methodology affects the association between detected PIK3CA mutations and PFS. In particular, PIK3CA mutations detected via ctDNA testing were more strongly prognostic of shorter PFS than mutations detected via tissue testing.

PI3Ks play a role in regulating multiple signaling pathways that are involved in cell proliferation, growth, survival, and other physiological functions and cellular processes [9]. Abnormal signaling through this pathway relates to several important aspects of cancer prognosis and treatment, including tumorigenesis, progression, and therapeutic resistance, which suggests prognostic relevance of PIK3CA mutations for patients with mBC [9–11]. Previous evidence on the role of PIK3CA mutations is varied and dependent on the specific patient subgroup. Among patients with early breast cancer, PIK3CA mutations were associated with better invasive disease-free survival, but not distant disease-free survival or overall survival [43]. Among patients with triple-negative breast cancer, evidence from a study with 119 patients seems to suggest a positive prognostic impact of a PIK3CA mutation [44]. In contrast, among patients with any type of breast cancer, PIK3CA was found to be a negative prognostic factor for survival outcomes comparing mutated vs wild-type PIK3CA patients in clinical trials [45]. A similar finding was noted for patients with HR + /HER2- mBC (the same patient group as in our study) who participated in the SAFIR02 (NCT02299999) trial, where patients with PIK3CA mutations were found to have worse outcomes than wild-type patients [46]. Our study’s finding agrees with those from the SAFIR02 trial (which also constituted a data point in our analysis), thus suggesting a consistent prognostic impact of PIK3CA in HR + /HER2- mBC.

In the current study, the prognostic impact of a PIK3CA mutation was estimated as an approximately 2 months shorter median PFS and 8 months shorter OS. This represents a clinically meaningful difference, relative to an overall median PFS of only 6 months or OS of 32 months among the included studies. The magnitude of the estimated effect sizes is similar to other mutations in mBC. For example, the BOLERO-2 clinical trial estimated that patients with mBC with an estrogen receptor 1 gene (D538G or Y537S) mutation had a 1 month shorter PFS (2.8 months vs 3.9 months) and 11 months shorter OS (20.7 vs 32.1) than patients without an estrogen receptor 1 mutation [47].

The magnitude of effect was consistent across patients treated with and without fulvestrant, and also similar upon adjustment for more granular classifications of treatments included in the present study which by design excluded PI3K-targeted therapies. This stands in contrast to the apparent association between mutation testing method and the prognostic value of detected PIK3CA mutations. This finding could be due to multiple hypotheses. For example, a previous study found differences in accuracy between PIK3CA mutation status determined via ctDNA testing and tissue testing, although the authors noted these findings could have been due to the use of archival tissue samples, which may not have correctly reflected mutation status at study entry [15]. Another possibility is the different nature of ctDNA testing, which may capture shed DNA from various metastatic sites, and detect disease progression and recurrence before other radiological procedures, compared to direct testing of the tissue [48–50]. However, in the current meta-analysis, a difference between testing methods in the prognostic relevance of PIK3CA was only detected when analyzing median PFS, but not for any of the PFS rate outcomes at 6, 12, or 18 months. In addition, the analyzed model only included the PIK3CA testing method as a moderating factor and this moderating relationship may have been confounded by the omission of other potentially important moderators. Thus, the association between testing type and prognostic value of PIK3CA warrants further confirmation before impacting the interpretation of specific testing methods.

The analyses of PIK3CA status and PFS rates at 6 and 12 months revealed similar prognostic associations as those seen with median PFS and median OS. Mutation was associated with an approximately 30% lower odds of remaining free of progression or death at these time points, and these associations were robust to adjustment for treatment type. At 18 months, data were sparse, and results were directionally consistent with similar magnitudes but less precision. Taken together, these findings indicate a negative prognostic value of PIK3CA mutation for both near- and longer-term PFS and OS in HR + /HER2- mBC.

Our findings provide a better understanding of the importance of PIK3CA in the prognosis of mBC and help make sense of conflicting evidence in the literature. In addition, our findings suggest that patients with PIK3CA-mutated mBC suffer from increased clinical burden and may particularly benefit from effective targeted therapies. Finally, clinicians may better inform their patients and families about the risk of death or recurrence using our findings.

This study is subject to a number of limitations, some of which are inherent to meta-analyses and meta-regression studies. Most importantly, meta-regressions can only account for confounding factors with sufficient data reported at the trial level. Studies with treatments that did not target PIK3CA mutations often did not report baseline characteristics stratified by PIK3CA mutation status, which would be required for adjustment in the present analysis. This means that potentially important confounders, such as performance status or proportions of patients with prior chemotherapy, were not sufficiently populated for adjustment in the meta-regression models [51]. The prognostic associations that we report should therefore be interpreted as inclusive of any average differences in baseline status between patients with mutated vs. wild-type PIK3CA. At the same time, our finding that heterogeneity due to cross-trial differences contributed at most a moderate part of the variation in estimated PIK3CA effects, as indicated by low to moderate I^2^ values, provides confidence that the estimated prognostic associations are meaningful and representative. Finally, data on OS was limited at the time of this analysis, so only an unadjusted model could be fit. However, the findings from this unadjusted model are consistent with the adjusted analyses in the PFS analyses and thus provide further evidence of a prognostic effect of a PIK3CA mutation.

Publication bias across studies was not formally assessed as the nature of this analysis likely precluded such concerns. Specifically, the included clinical trials aimed to establish a significant treatment effect comparing patients in treatment and control arms. However, the present study was interested in estimating differences between patients within treatment or control arms across mutation cohorts, which was orthogonal to the publication interest of the included studies. In addition, data from clinical trials are less likely subject to publication bias due to the mandatory registration requirements.

Future studies may collect additional data from upcoming trials and further study overall survival and additional confounders that the current study was not able to fully control for.

## Conclusion

Among patients with HR + /HER2- mBC who are receiving therapies that do not target PIK3CA, a mutation of PIK3CA is a negative prognostic factor, associated with significantly shorter PFS by approximately 2 months and shorter OS by approximately 8 months. These findings highlight the increased clinical burden of PIK3CA-mutated mBC, and the importance of effective therapies for this population. 

## Supplementary Information

**Additional file 1.** 

## Data Availability

The datasets used and/or analyzed during the current study available from the corresponding author on reasonable request.

## Abbreviations

BC: Breast cancer
CI: Confidence intervals
ctDNA: Circulating tumor DNA
HER2 +: Human epidermal growth factor receptor 2 positive
HR +: Hormone receptor positive
KM: Kaplan–Meier
mBC: Metastatic breast cancer
OS: Overall survival
PFS: Progression-free survival
PIK3CA: Phosphatidylinositol-4,5-bisphosphate 3-kinase catalytic subunit alpha
REML: Restricted maximum likelihood
US: United States
