# Supplementary material for: PIK3CA mutation status, progression and survival in advanced HR + /HER2- breast cancer: a meta-analysis of published clinical trials
Source: BMC Cancer. 2022 Sep 21;22:1002. doi: 10.1186/s12885-022-10078-5 (PMC9490901; doi:10.1186/s12885-022-10078-5)
Supplement: Supplementary file 1 — Additional file 1. [file 12885_2022_10078_MOESM1_ESM.docx]

PIK3CA mutation status, progression and survival in advanced HR+/HER2- breast cancer: a meta-analysis of published clinical trials

**Authors**: Mirko Fillbrunn^1^, James Signorovitch^1^, Fabrice Andre^2^, Iris Wang^3^, Ines Lorenzo^4^, Antonia Ridolfi^5^, Jinhee Park^3^, Akanksha Dua^1^, Hope Rugo^6^

**Affiliations:**

1. Analysis Group, Inc., Boston, MA, USA
2. Institut Gustave Roussy, Villejuif, France
3. Novartis, East Hanover, NJ, USA
4. Novartis, Madrid, Spain
5. Novartis, Paris, France
6. University of California San Francisco, Helen Diller Family Comprehensive Cancer Center, San Francisco, California, USA

**Corresponding Author: James Signorovitch**

Mailing address: Analysis Group, Inc.,

111 Huntington Avenue, 14^th^ Floor, Boston, MA- 02199

Phone: (617) 425 8258

Email: James.Signorovitch@analysisgroup.com

[1. Abstract Screening Tool for Epidemiology and Testing Methods of PIK3CA 3](#_Toc47444569)

[2. Complete search strategy by data base 7](#_Toc47444570)

[3. Technical details for time-to-event curve extraction from publications 12](#_Toc47444571)

[4. Data availability by publication 14](#_Toc47444572)

[Table S1. Availability of selected outcomes and study/baseline characteristics by publication^1^ 14](#_Toc47444573)

[5. Detailed tables of meta-regressions 16](#_Toc47444574)

[Table S2. Meta-regression results for PFS median; models without interactions 16](#_Toc47444575)

[Table S3. Meta-regression results for PFS median; models with interactions 19](#_Toc47444576)

[Table S4. Meta-regression results for 6-month PFS rates; models without interactions 23](#_Toc47444577)

[Table S5. Meta-regression results for 6-month PFS rates; models with interactions 27](#_Toc47444578)

[Table S6. Meta-regression results for 12-month PFS rates; models without interactions 31](#_Toc47444579)

[Table S7. Meta-regression results for 12-month PFS rates; models with interactions 34](#_Toc47444580)

[Table S8. Meta-regression results for 18-month PFS rates; models without interactions 38](#_Toc47444581)

[Table S9. Meta-regression results for 18-month PFS rates; models with interactions 41](#_Toc47444582)

[Figure S1. Within-study differences between PIK3CA-mutated and wild-type cohorts (a) 12-month PFS rate (odds ratio) (b) 18-month PFS rate (odds ratio) 46](#_Toc47444583)

[6. References 47](#_Toc47444584)

# Abstract Screening Tool for Epidemiology and Testing Methods of PIK3CA

The following material were used in the systematic literature review that informed our study selection.^1^

**Study Abstract Screening Tool**

**A systematic literature review of the epidemiology and the clinical, economic, and humanistic burden for postmenopausal women with PI3KCA-mutant HR+/HER2-negative advanced or metastatic breast cancer**

Unique Identifier: Reviewer Initials:

Author, Year: Review Date:

Utilize the definition table located at the end of this document to ensure questions are answered correctly. Please review the abstract and answer the following questions for inclusion.

*(If unable to determine the answers from the abstract or no abstract is available, use the full text article for review)*

|  | Question | Answer | Directions |
| --- | --- | --- | --- |
| Q1 | Does the study include information on: **the epidemiology or testing methods of PIK3CA mutations in women with advanced or metastatic HR+, HER2- breast cancer?** |  |  |
|  |  |  |  |
| 1a. | **the epidemiology or testing methods of PIK3CA mutations** | ☐ Yes | Continue to Q1b. |
|  |  | ☐ No |  |
| 1b. | advanced or metastatic | ☐ Yes | Continue to Q1c. |
|  |  | ☐ No | If no, STOP – study excluded |
| 1c. | HR+, HER2- breast cancer | ☐ Yes | Continue to question 2. |
|  |  | ☐ No | STOP – study excluded |
| Q2 | Does the study examine **and identify differences between PIK3CA mutant and wild-type populations?** | ☐ Yes | Continue to question 3 |
|  |  | ☐ No | STOP – study excluded |
| Q3 | Does this study describe its biopsy testing method? | ☐ Yes | Continue to question 4 |
|  |  | ☐ No | STOP – study excluded |
| Q4 | Does this study report performance measures or outcomes? |  |  |
| 4a. | Performance Measures | ☐ Yes | Continue to question 4b. |
|  |  | ☐ No |  |
| 4b. | Outcomes | ☐ Yes | If NO to BOTH 4a and 4b, STOP-study excluded.  Otherwise continue to exclusion criteria |
|  |  | ☐ No |  |

HER2-, human epidermal growth factor receptor 2 negative; HR+, hormone receptor positive; PIK3CA, phosphatidylinositol-4,5-bisphosphate 3-kinase catalytic subunit alpha

______________________________________________________________________________

Note any additional reason(s) for exclusion below. (Check all that apply)

☐ Editorial/Letter

☐ Commentary

☐ Review

☐ Other:

☐ Not in humans

☐ Not in English language

☐ Date out of range (before 2000)

______________________________________________________________________________

Date of review team meeting:

Decision: ☐ Include ☐ Exclude ☐ Arbitration Needed

Date of arbitration team meeting:

Decision: ☐ Include ☐ Exclude

| Term | Criteria |
| --- | --- |
| Currently available therapies | Any therapy currently utilized for the treatment of HR+/HER2-negative advanced or metastatic breast cancer |
| PIK3CA mutant populations | Patients with PIK3CA mutation |
| PIK3CA wild-type populations | Patients without PIK3CA |
| Sensitivity | Ability of test of interest to correctly identify a positive result |
| Specificity | Ability of test of interest to correctly identify a negative result |
| Prevalence | Prevalence of PIK3CA mutation in HR+/HER2- advanced or metastatic breast cancer post-menopausal women |
| Frequency of testing | Number of times test is repeated within the course of interaction with the participant |

HER2-, human epidermal growth factor receptor 2 negative; HR+, hormone receptor positive; PIK3CA, phosphatidylinositol-4,5-bisphosphate 3-kinase catalytic subunit alpha

# Complete search strategy by data base

Search strategy

The following search strategies will be used, including all the search terms: text words (free text), subject index headings (for example, MeSH) and the relationship between the search terms (for example, Boolean).

Terms may be enriched by other synonyms not otherwise specified here.

**MEDLINE, MEDLINE (R) In-Process, EMBASE CDSR, DARE and CENTRAL (Ovid)**

| **No.** | **Search String** |
| --- | --- |
| 1 | exp breast neoplasms/ or exp breast cancer/ |
| 2 | (breast$ adj3 (cancer$ or neoplas$ or oncolog$ or tumo?r$ or malignanc$ or carcinoma$ or adenocarcinoma$ or sarcoma$)).ti,ab. |
| 3 | (mammar$ adj3 (cancer$ or neoplas$ or oncolog$ or tumo?r$ or malignanc$ or carcinoma$ or adenocarcinoma$ or sarcoma$)).ti,ab. |
| 4 | (metasta$ or advance$ or second$ or recurren$ or inoperab$ or disseminat$ or incur$).ti,ab,sh. |
| 5 | (1 or 2 or 3) and 4 |
| 6 | exp Breast/ and exp Neoplasm Metastasis/ |
| 7 | (breast$ adj3 (metasta$ or advance$ or second$ or recurren$ or inoperab$ or disseminat$ or incur$)).ti,ab. |
| 8 | (mammar$ adj3 (metasta$ or advance$ or second$ or recurren$ or inoperab$ or disseminat$ or incur$)).ti,ab. |
| 9 | (breast$ or mammar$).ti,ab,sh. |
| 10 | ((stage or grade or type) adj2 ("3" or III or "c" or "4" or "IV" or d)).ti,ab. |
| 11 | (N1 or N2$ or N3$ or pN1$ or pN2$ or pN3$).ti,ab,sh. |
| 12 | 9 and (10 or 11) |
| 13 | OR/5-8,12 |
| 14 | (animals not humans).sh. |
| 15 | (comment or editorial or or editorial or book or practice-guideline or letter or journal correspondence).pt. |
| 16 | NOT (14 or 15) |
| 17 | 13 and 16 |
| 18 | (Phosphatidylinositol 3-Kinases or “PIK3CA protein, human”) |
| 19 | 17 and 18 |
| 20 | limit 19 to english |
| 21 | remove duplicates from 20 |

**PUBMED Search Strategy**

("PIK3CA protein, human"[Supplementary Concept] OR "Phosphatidylinositol 3-Kinases"[Mesh] OR “PIK3CA”[ALL] OR “Phosphatidylinositol 3-Kinases”[ALL]) AND ("Breast Neoplasms"[Mesh] OR "Unilateral Breast Neoplasms"[Mesh] OR "Breast Cancer 3"[Supplementary Concept] OR "Breast Cancer, Familial"[Supplementary Concept] OR "estrogen regulated protein"[Supplementary Concept] OR “breast cancer”[ALL] OR “breast neoplasm*”[ALL])

**ClinicalTrials.gov**

(www.clinicaltrials.gov)

Search terms:

(letrozole OR Femara) AND (breast OR mammar*)

(anastrozole OR Arimidex) AND (breast OR mammar*)

(exemestane or Aromasin or Aromasil) AND (breast OR mammar*)

(tamoxifen OR Nolvadex OR Soltamox) AND (breast OR mammar*)

(fulvestrant OR Faslodex) AND (breast OR mammar*)

(palbociclib OR Ibrance) AND (breast OR mammar*)

(everolimus OR afinitor ) AND (breast OR mammar*)

(LEE011 OR LEE-011 OR Ribociclib) AND (breast OR mammar*)

(abemaciclib) AND (breast OR mammar*)

(capecitabine OR Xeloda) AND (breast OR mammar*)

(doxorubicin OR Adriamycin OR Doxil) AND (breast OR mammar*)

(paclitaxel OR Abraxane) AND (breast OR mammar*)

(ethinyl estradiol) AND (breast OR mammar*)

((Fluoxymesterone OR Stenox OR Halotestin OR fluoximesterone) AND (breast OR mammar*)

(megestrol acetate OR Megace OR Mestrel OR Maygace OR Megostat) AND (breast OR mammar*)

(docetaxel OR Taxotere OR Docefrez) AND (breast OR mammar*)

(cyclophosphamide OR cytophosphane OR Cytoxan OR Endoxan ) AND (breast OR mammar*)

(eribulin OR Halaven) AND (breast OR mammar*)

(methotrexate OR Trexall) AND (breast OR mammar*)

(fluorouracil OR Adrucil OR Fluorouracil Novaplus OR PremierPro Rx Fluorouracil) AND (breast OR mammar*)

(toremifene OR Fareston) AND (breast OR mammar*)

(gemcitabine OR Gemcitabine Novaplus OR Gemzar OR PremierPro Rx Gemcitabine) AND (breast OR mammar*)

(vinorelbine OR Navelbine OR Vinorelbine Novaplus) AND (breast OR mammar*)

(paclitaxel OR paclitaxel protein bound OR Taxol OR Abraxane) AND (breast OR mammar*)

(ixabepilone OR Ixempra) AND (breast OR mammar*)

(cisplatin OR Platinol-AQ) AND (breast OR mammar*)

(carboplatin OR Paraplatin OR Paraplatin NovaPlus OR ) AND (breast OR mammar*)

(next-generation sequencing) AND (breast OR mammar*) AND (PIK3CA)

(tumor biopsy) AND (breast OR mammar*) AND (PIK3CA)

(liquid biopsies) AND (breast OR mammar*) AND (PIK3CA)

(testing) AND (PIK3CA) AND (breast OR mammar*)

Abstracts from the following conferences will be searched, while other conferences of interest are covered by EMBASE.

**AACR 2016**

(http://www.abstractsonline.com/plan/AdvancedSearch.aspx)

Search terms:

Session Type: ALL

Session Title: ALL

Sponsored Sessions: ALL

Abstract Organ Site: Breast Cancer

Title/Abstract Body Text: “hormone receptor positive” OR “HR+” OR “ER+” OR “PR+” OR “estrogen receptor positive”

**ASCO Breast Cancer Symposium 2015**

(http://meetinglibrary.asco.org)

Search terms:

keywords: breast cancer AND (“hormone receptor positive” OR “HR+” OR “ER+” OR “PR+” OR “estrogen receptor positive”)

**SABCS 2016**

(http://www.sabcs.org/)

Search terms: breast cancer AND (“hormone receptor positive” OR “HR+” OR “ER+” OR “PR+” OR “estrogen receptor positive”)

**ECCO 2015-2016 and EBCC 2014, 2016**

(www.ecco-org.eu/)

Search terms: breast cancer AND (“hormone receptor positive” OR “HR+” OR “ER+” OR “PR+” OR “estrogen receptor positive”)

Abstract title: breast cancer

**ESMO 2014**

(http://annonc.oxfordjournals.org/)

Search strategy (via “Find articles in this issue containing these words” box):

Search terms:breast cancer AND (“hormone receptor positive” OR “HR+” OR “ER+” OR “PR+” OR “estrogen receptor positive”)

Abstract title: breast cancer

**Citation/Reference Search**

In addition, references and citations of included studies will also be searched for potential literature.

# Technical details for time-to-event curve extraction from publications

Time-to-event outcomes were reconstructed based on published Kaplan-Meier curves using digitization software. Specifically, the ENGAUGE digitization software (http://digitizer.sourceforge.net/) was used to extract survival probabilities at multiple time points from the published KM curves. Based on the extracted survival curves, and reported numbers of events and patients at risk at various time points in the publication, approximate individual patient data (pseudo-IPD) were generated using the approach developed by Guyot et al.^2^ The accuracy of the reconstructed data was assessed through comparison with published statistics on survival probabilities and median survival. This approach is consistent with NICE guidance.^3^ The pseudo-IPD generated from the PFS KM curves was then used to generate the standard errors of the median PFS via percentile bootstrap and the 6, 12, and 18 month PFS rates using the Greenwood formula.

When KM curves were not reported, standard errors (SEs) of median PFS were approximated using available extracted data (such as median, sample size, etc.) assuming an underlying exponential distribution of time-to-event outcomes. The consistency of the two methods to impute standard errors of median PFS were compared where both were available and found to have good consistency.

For Figures 2 a and b in the main manuscript and Figure S1, 95% confidence intervals of differences in PFS outcomes between PIK3CA-mutated and wild-type cohorts within study arms were computed using the pseudo-IPD via percentile bootstrap. These differences could only be computed where KM curves from both cohorts were available, so the differences shown include only a subset of the studies that were included in the meta-regressions.

Median follow-up time was imputed using the pseudo-IPD via a time to censoring approach if it was not directly reported in the trial.^4^

# Data availability by publication

## Table S1. Availability of selected outcomes and study/baseline characteristics by publication^1^

| **First Author, year** | **Study acronym** | **Sample size^2^** | | **Study/baseline characteristics** | | | | | **Outcomes^3^** | | | |
| --- | --- | --- | --- | --- | --- | --- | --- | --- | --- | --- | --- | --- |
|  |  | **Wild** | **Mutant** | **Follow-up time** | **Prior fulvestrant** | **Tissue/ ctDNA testing** | **Current treatment** | **% with prior chemotherapy** | **Median PFS** | **PFS KM curve** | **Median OS** | **OS KM curve** |
|  |  |  |  |  |  |  |  |  |  |  |  |  |
| Andre, 2021^5^ | SOLAR-1 | 116 | 172 | Yes | Yes | Yes | Yes | Yes | Yes | Yes | No | No |
| Juric, 2019^6^ | SOLAR-1 | 182 | 94 | Yes | Yes | Yes | Yes | Yes | Yes | Yes | Yes | Yes |
| Baselga, 2017^7^ | BELLE-2 | 188 | 113 | Yes | Yes | Yes | Yes | Yes | Yes | Yes | No | No |
| Campone, 2018^8^ | BELLE-2 | 188 | 113 | Yes | Yes | Yes | Yes | Yes | Yes | No | Yes | No |
| Cristofanilli, 2016^9^ | PALOMA-3 | 266 | 129 | Yes | Yes | Yes | Yes | Yes | Yes | No | No | No |
| Turner, 2018^10^ | PALOMA-3 | 266 | 129 | Yes | Yes | Yes | Yes | No | No | No | Yes | No |
| Di Leo, 2018^11^ | BELLE-3 | 150 | 69 | Yes | Yes | Yes | Yes | No | Yes | Yes | No | No |
| Dickler, 2018^12^ |  |  |  |  |  |  |  |  |  |  |  |  |
| Fleming, 2012^13^ |  | 19 | 5 | No | Yes | Yes | Yes | No | Yes | No | No | No |
| Hortobagyi, 2016^14^ | BOLERO-2 | 159 | 143 | Yes | Yes | Yes | Yes | No | Yes | Yes | No | No |
| Krop, 2016^15^ | FERGI | 39 | 32 | Yes | Yes | Yes | Yes | No | Yes | Yes | No | No |
| Mayer, 2014^16^ |  |  |  |  |  |  |  |  |  |  |  |  |
| Mayer, 2017^16^ |  |  |  |  |  |  |  |  |  |  |  |  |
| Moynahan, 2017^17^ | BOLERO-2 | 312 | 238 | Yes | Yes | Yes | Yes | No | Yes | Yes | No | No |
| Mosele, 2019^18^ | SAFIR02_Breast | 260 | 104 | Yes | Yes | Yes | Yes | No | No | Yes | Yes | No |
| Baselga, 2018^19^ | SANDPIPER | 38 | 176 | Yes | Yes | Yes | Yes | No | Yes | Yes | No | No |
| Tolaney, 2022^20^ | MONARCH 2 | 133 | 86 | Yes | Yes | Yes | Yes | No | Yes | Yes | Yes | Yes |

ctDNA, circulating tumor deoxyribose nucleic acid; KM, Kaplan-Meier; PIK3CA, phosphatidylinositol-4,5-bisphosphate 3-kinase catalytic subunit alpha; PFS, progression-free survival, OS, survival

**Notes**:

[1] Table presents data only on outcomes and study characteristics that were included in the analysis. Availability is based on whether statistics were reported in the study by PIK3CA mutation subgroup. Individual studies may have individual treatment arms excluded from analysis due to the use of PIK3CA-targeted treatment.

[2] Sample sizes may differ based on analysis specification. Missing sample sizes in publications were filled in from other publications of the same clinical trial.

[3] Outcomes and follow-up time availability accounts for where data were approximated based on KM curves.

# Detailed tables of meta-regressions

The following regression tables include the analyses presented in the main article.

## Table S2. Meta-regression results for PFS median; models without interactions

Association of PIK3CA mutation with median PFS (months): Base model

| **Variable** | **Estimate** | **SE** | **CI-Lower** | **CI-Upper** | **P-value** |
| --- | --- | --- | --- | --- | --- |
| Intercept | 7.98 | 2.15 | 3.59 | 12.38 | P < 0.001 *** |
| PIK3CA Status Mutated | -1.78 | 0.81 | -3.44 | -0.13 | P < 0.05 * |
| Patients (mutated, wild)=3219 (1386, 1833) | Study arms=33, Pubs.=11 | AIC=264.13 | Q=486.93 | Q p-value=0.00 | I2=34.51% |
| *Note:* |  |  |  |  |  |
| Signif. codes: 0.001 " *** " 0.01 " ** " 0.05 " * " |  |  |  |  |  |

Base model + controlling for ctDNA testing methodology

| **Variable** | **Estimate** | **SE** | **CI-Lower** | **CI-Upper** | **P-value** |
| --- | --- | --- | --- | --- | --- |
| Intercept | 5.96 | 0.98 | 3.96 | 7.96 | P < 0.001 *** |
| PIK3CA Status Mutated | -1.28 | 0.69 | -2.69 | 0.12 | P = 0.07 |
| ctDNA Testing Tissue testing | -0.24 | 0.37 | -0.99 | 0.51 | P = 0.51 |
| Patients (mutated, wild)=2855 (1282, 1573) | Study arms=31, Pubs.=10 | AIC=241.41 | Q=313.76 | Q p-value=0.00 | I2=29.81% |
| Note: |  |  |  |  |  |
| Signif. codes: 0.001 " *** " 0.01 " ** " 0.05 " * " |  |  |  |  |  |

Base model + controlling for fulvestrant treatment

| **Variable** | **Estimate** | **SE** | **CI-Lower** | **CI-Upper** | **P-value** |
| --- | --- | --- | --- | --- | --- |
| Intercept | 12.98 | 2.43 | 8.01 | 17.95 | P < 0.001 *** |
| PIK3CA Status Mutated | -1.83 | 0.54 | -2.93 | -0.73 | P < 0.01 ** |
| Fulvestrant Treatment Yes | -6.40 | 1.98 | -10.44 | -2.36 | P < 0.01 ** |
| Patients (mutated, wild)=3219 (1386, 1833) | Study arms=33, Pubs.=11 | AIC=258.93 | Q=448.90 | Q p-value=0.00 | I2=36.23% |
| Note: |  |  |  |  |  |
| Signif. codes: 0.001 " *** " 0.01 " ** " 0.05 " * " |  |  |  |  |  |

Base model + controlling for study treatment

| **Variable** | **Estimate** | **SE** | **CI-Lower** | **CI-Upper** | **P-value** |
| --- | --- | --- | --- | --- | --- |
| Intercept | 15.81 | 4.67 | 6.22 | 25.41 | P < 0.01 ** |
| PIK3CA Status Mutated | -1.25 | 0.45 | -2.18 | -0.33 | P < 0.01 ** |
| Treatment Everolimus + Exemestane | -7.52 | 4.87 | -17.54 | 2.50 | P = 0.13 |
| Treatment Palbociclib + Fulvestrant | -5.23 | 4.75 | -14.99 | 4.54 | P = 0.28 |
| Treatment Placebo + Exemestane | -11.67 | 4.88 | -21.70 | -1.64 | P < 0.05 * |
| Treatment Placebo + Fulvestrant | -5.23 | 4.75 | -14.99 | 4.54 | P = 0.28 |
| Treatment Other | 6.55 | 5.16 | -4.06 | 17.16 | P = 0.22 |
| Patients (mutated, wild)=3219 (1386, 1833) | Study arms=33, Pubs.=11 | AIC=142.49 | Q=98.10 | Q p-value=0.00 | I2=33.33% |
| Note: |  |  |  |  |  |
| Signif. codes: 0.001 " *** " 0.01 " ** " 0.05 " * " |  |  |  |  |  |
| 1 Other treatment arm may include targeted therapy, standard maintanance chemotherapy or immunotherapy. |  |  |  |  |  |

AIC, Akaike Information Criterion; CI, confidence interval; ctDNA, circulating tumor deoxyribose nucleic acid; I^2^ , I^2^ statistic in %; PIK3CA, phosphatidylinositol-4,5-bisphosphate 3-kinase catalytic subunit alpha; PFS, progression-free survival; Q, Test statistic for (residual) heterogeneity, Q p-value, p-value for test for (residual) heterogeneity; SE, standard error

**Notes:**
[1] All estimates provided represent months.
[2] All models included random effects at the study and publication level.

## Table S3. Meta-regression results for PFS median; models with interactions

Base model + controlling for ctDNA testing and interaction with PIK3CA status

| **Variable** | **Estimate** | **SE** | **CI-Lower** | **CI-Upper** | **P-value** |
| --- | --- | --- | --- | --- | --- |
| ntercept | 6.38 | 0.88 | 4.57 | 8.19 | P < 0.001 *** |
| PIK3CA Status Mutated | -1.86 | 0.57 | -3.03 | -0.70 | P < 0.01 ** |
| ctDNA Testing Tissue testing | -1.51 | 0.64 | -2.83 | -0.19 | P < 0.05 * |
| PIK3CA Status Mutated:ctDNA Testing Tissue testing | 1.74 | 0.66 | 0.39 | 3.09 | P < 0.05 * |
| Patients (mutated, wild)=2855 (1282, 1573) | Study arms=31, Pubs.=10 | AIC=238.35 | Q=302.94 | Q p-value=0.00 | I2=37.1% |
| Note: |  |  |  |  |  |
| Signif. codes: 0.001 " *** " 0.01 " ** " 0.05 " * " |  |  |  |  |  |

[1] “PIK3CA status association among studies with tissue testing: -0.13 [-1.36,1.11]”

Base model + controlling for fulvestrant treatment and interaction with PIK3CA status

| **Variable** | **Estimate** | **SE** | **CI-Lower** | **CI-Upper** | **P-value** |
| --- | --- | --- | --- | --- | --- |
| Intercept | 13.75 | 4.18 | 5.21 | 22.30 | P < 0.01 ** |
| PIK3CA Status Mutated | -2.50 | 1.31 | -5.17 | 0.18 | P = 0.07 |
| Fulvestrant Treatment Yes | -7.33 | 4.74 | -17.03 | 2.36 | P = 0.13 |
| PIK3CA Status Mutated:Fulvestrant Treatment Yes | 0.64 | 1.51 | -2.46 | 3.73 | P = 0.68 |
| Patients (mutated, wild)=3219 (1386, 1833) | Study arms=33, Pubs.=11 | AIC=261 | Q=440.05 | Q p-value=0.00 | I2=33.19% |
| Note: |  |  |  |  |  |
| Signif. codes: 0.001 " *** " 0.01 " ** " 0.05 " * " |  |  |  |  |  |

Base model + controlling for study treatment and interaction with PIK3CA status

| **Variable** | **Estimate** | **SE** | **CI-Lower** | **CI-Upper** | **P-value** |
| --- | --- | --- | --- | --- | --- |
| Intercept | 13.83 | 8.10 | -3.01 | 30.68 | P = 0.10 |
| PIK3CA Status Mutated | 1.74 | 9.70 | -18.44 | 21.92 | P = 0.86 |
| Treatment Everolimus + Exemestane | -6.08 | 8.41 | -23.57 | 11.40 | P = 0.48 |
| Treatment Palbociclib + Fulvestrant | -3.36 | 8.18 | -20.37 | 13.65 | P = 0.69 |
| Treatment Placebo + Exemestane | -10.30 | 8.38 | -27.74 | 7.13 | P = 0.23 |
| Treatment Placebo + Fulvestrant | -8.50 | 8.10 | -25.35 | 8.36 | P = 0.31 |
| Treatment Other | 9.62 | 8.54 | -8.15 | 27.38 | P = 0.27 |
| PIK3CA Status Mutated interacted with Treatment Everolimus + Exemestane | -2.69 | 9.83 | -23.13 | 17.75 | P = 0.79 |
| PIK3CA Status Mutated interacted with Treatment Palbociclib + Fulvestrant | -2.44 | 9.85 | -22.92 | 18.04 | P = 0.81 |
| PIK3CA Status Mutated interacted with Treatment Placebo + Exemestane | -2.55 | 9.81 | -22.95 | 17.85 | P = 0.80 |
| PIK3CA Status Mutated interacted with Treatment Placebo + Fulvestrant | -3.21 | 9.72 | -23.42 | 17.01 | P = 0.74 |
| PIK3CA Status Mutated interacted with Treatment Other | -6.16 | 10.31 | -27.61 | 15.29 | P = 0.56 |
| Patients (mutated, wild)=3219 (1386, 1833) | Study arms=33, Pubs.=11 | AIC=152.69 | Q=95.19 | Q p-value=0.00 | I2=27.44% |
| Note: |  |  |  |  |  |
| Signif. codes: 0.001 " *** " 0.01 " ** " 0.05 " * " |  |  |  |  |  |
| 1 Other treatment arm may include targeted therapy, standard maintanance chemotherapy or immunotherapy. |  |  |  |  |  |

AIC, Akaike Information Criterion; CI, confidence interval; ctDNA, circulating tumor deoxyribose nucleic acid; PIK3CA, phosphatidylinositol-4,5-bisphosphate 3-kinase catalytic subunit alpha; PFS, progression-free survival; Q, Test statistic for (residual) heterogeneity, Q p-value, p-value for test for (residual) heterogeneity, I^2^ , I^2^ statistic in %; SE, standard error

**Notes:**
[1] All estimates provided represent months.
[2] All models included random effects at the study and publication level.

## Table S4. Meta-regression results for 6-month PFS rates; models without interactions

Association of PIK3CA mutation with 6 month survival rate (odds ratio): Base model

| **Variable** | **Estimate** | **SE** | **CI-Lower** | **CI-Upper** | **P-value** |
| --- | --- | --- | --- | --- | --- |
| Intercept | 1.32 | 1.44 | 0.62 | 2.79 | P = 0.46 |
| PIK3CA Status Mutated | 0.74 | 1.12 | 0.59 | 0.94 | P < 0.05 * |
| Patients (mutated, wild)=3160 (1366, 1794) | Study arms=31, Pubs.=11 | AIC=144 | Q=274.44 | Q p-value=0.00 | I2=41.68% |
| Note: |  |  |  |  |  |
| Signif. codes: 0.001 " *** " 0.01 " ** " 0.05 " * " |  |  |  |  |  |

Base model + controlling for ctDNA testing methodology

| **Variable** | **Estimate** | **SE** | **CI-Lower** | **CI-Upper** | **P-value** |
| --- | --- | --- | --- | --- | --- |
| Intercept | 0.94 | 1.31 | 0.55 | 1.63 | P = 0.83 |
| PIK3CA Status Mutated | 0.80 | 1.15 | 0.60 | 1.07 | P = 0.13 |
| ctDNA Testing Tissue testing | 1.10 | 1.12 | 0.87 | 1.39 | P = 0.42 |
| Patients (mutated, wild)=2796 (1262, 1534) | Study arms=29, Pubs.=10 | AIC=132.61 | Q=189.67 | Q p-value=0.00 | I2=26.69% |
| Note: |  |  |  |  |  |
| Signif. codes: 0.001 " *** " 0.01 " ** " 0.05 " * " |  |  |  |  |  |

Base model + controlling for fulvestrant treatment

| **Variable** | **Estimate** | **SE** | **CI-Lower** | **CI-Upper** | **P-value** |
| --- | --- | --- | --- | --- | --- |
| Intercept | 4.07 | 1.80 | 1.21 | 13.63 | P < 0.05 * |
| PIK3CA Status Mutated | 0.72 | 1.11 | 0.59 | 0.90 | P < 0.01 ** |
| Fulvestrant Treatment Yes | 0.24 | 1.86 | 0.07 | 0.86 | P < 0.05 * |
| Patients (mutated, wild)=3160 (1366, 1794) | Study arms=31, Pubs.=11 | AIC=140.83 | Q=271.86 | Q p-value=0.00 | I2=40.43% |
| Note: |  |  |  |  |  |
| Signif. codes: 0.001 " *** " 0.01 " ** " 0.05 " * " |  |  |  |  |  |

Base model + controlling for study treatment

| **Variable** | **Estimate** | **SE** | **CI-Lower** | **CI-Upper** | **P-value** |
| --- | --- | --- | --- | --- | --- |
| Intercept | 1.76 | 1.44 | 0.83 | 3.74 | P = 0.13 |
| PIK3CA Status Mutated | 0.71 | 1.10 | 0.59 | 0.86 | P < 0.01 ** |
| Treatment Everolimus + Exemestane | 0.97 | 1.94 | 0.25 | 3.85 | P = 0.97 |
| Treatment Palbociclib + Fulvestrant | 1.63 | 1.46 | 0.74 | 3.58 | P = 0.21 |
| Treatment Placebo + Exemestane | 0.25 | 1.97 | 0.06 | 1.01 | P = 0.05 |
| Treatment Placebo + Fulvestrant | 0.48 | 1.36 | 0.25 | 0.90 | P < 0.05 * |
| Treatment Other | 8.06 | 2.06 | 1.81 | 35.87 | P < 0.01 ** |
| Patients (mutated, wild)=3160 (1366, 1794) | Study arms=31, Pubs.=11 | AIC=45.72 | Q=65.20 | Q p-value=0.00 | I2=43.46% |
| Note: |  |  |  |  |  |
| Signif. codes: 0.001 " *** " 0.01 " ** " 0.05 " * " |  |  |  |  |  |
| ^1^ Other treatment arm may include targeted therapy, standard maintenance chemotherapy or immunotherapy. |  |  |  |  |  |

AIC, Akaike Information Criterion; CI, confidence interval; ctDNA, circulating tumor deoxyribose nucleic acid; PIK3CA, phosphatidylinositol-4,5-bisphosphate 3-kinase catalytic subunit alpha; PFS, progression-free survival; Q, Test statistic for (residual) heterogeneity, Q p-value, p-value for test for (residual) heterogeneity, I^2^ , I^2^ statistic in %; SE, standard error

**Notes:**
[1] All estimates provided represent odds ratios surviving at 6 months.
[2] All models included random effects at the study and publication level.

## Table S5. Meta-regression results for 6-month PFS rates; models with interactions

Base model + controlling for ctDNA testing and interaction with PIK3CA status

| **Variable** | **Estimate** | **SE** | **CI-Lower** | **CI-Upper** | **P-value** |
| --- | --- | --- | --- | --- | --- |
| Intercept | 0.98 | 1.29 | 0.58 | 1.67 | P = 0.95 |
| PIK3CA Status Mutated | 0.72 | 1.15 | 0.53 | 0.96 | P < 0.05 * |
| ctDNA Testing Tissue testing | 0.93 | 1.19 | 0.65 | 1.33 | P = 0.67 |
| PIK3CA Status Mutated interacted with ctDNA Testing Tissue testing | 1.33 | 1.25 | 0.84 | 2.12 | P = 0.22 |
| Patients (mutated, wild)=2796 (1262, 1534) | Study arms=29, Pubs.=10 | AIC=133.35 | Q=185.98 | Q p-value=0.00 | I2=33.55% |
| Note: |  |  |  |  |  |
| Signif. codes: 0.001 " *** " 0.01 " ** " 0.05 " * " |  |  |  |  |  |

[1] “PIK3CA status association among studies with tissue testing: -0.05 [-0.43,0.34]”

Base model + controlling for fulvestrant treatment and interaction with PIK3CA status

| **Variable** | **Estimate** | **SE** | **CI-Lower** | **CI-Upper** | **P-value** |
| --- | --- | --- | --- | --- | --- |
| Intercept | 3.66 | 2.01 | 0.87 | 15.37 | P = 0.07 |
| PIK3CA Status Mutated | 0.76 | 1.21 | 0.52 | 1.12 | P = 0.16 |
| Fulvestrant Treatment Yes | 0.28 | 2.22 | 0.05 | 1.41 | P = 0.12 |
| PIK3CA Status Mutated interacted with Fulvestrant Treatment Yes | 0.93 | 1.26 | 0.58 | 1.50 | P = 0.76 |
| Patients (mutated, wild)=3160 (1366, 1794) | Study arms=31, Pubs.=11 | AIC=143.01 | Q=271.54 | Q p-value=0.00 | I2=40.89% |
| Note: |  |  |  |  |  |
| Signif. codes: 0.001 " *** " 0.01 " ** " 0.05 " * " |  |  |  |  |  |

Base model + controlling for study treatment and interaction with PIK3CA status

| **Variable** | **Estimate** | **SE** | **CI-Lower** | **CI-Upper** | **P-value** |
| --- | --- | --- | --- | --- | --- |
| Intercept | 1.32 | 1.57 | 0.51 | 3.41 | P = 0.55 |
| PIK3CA Status Mutated | 1.39 | 1.66 | 0.48 | 4.00 | P = 0.52 |
| Treatment Everolimus + Exemestane | 1.20 | 2.23 | 0.22 | 6.42 | P = 0.82 |
| Treatment Palbociclib + Fulvestrant | 2.36 | 1.62 | 0.86 | 6.46 | P = 0.09 |
| Treatment Placebo + Exemestane | 0.32 | 2.25 | 0.06 | 1.75 | P = 0.18 |
| Treatment Placebo + Fulvestrant | 0.63 | 1.49 | 0.27 | 1.45 | P = 0.26 |
| Treatment Other | 10.73 | 2.36 | 1.78 | 64.71 | P < 0.05 * |
| PIK3CA Status Mutated interacted with Treatment Everolimus + Exemestane | 0.56 | 1.94 | 0.14 | 2.23 | P = 0.39 |
| PIK3CA Status Mutated interacted with Treatment Palbociclib + Fulvestrant | 0.39 | 1.83 | 0.11 | 1.40 | P = 0.14 |
| PIK3CA Status Mutated interacted with Treatment Placebo + Exemestane | 0.51 | 2.03 | 0.11 | 2.24 | P = 0.35 |
| PIK3CA Status Mutated interacted with Treatment Placebo + Fulvestrant | 0.55 | 1.66 | 0.19 | 1.60 | P = 0.26 |
| PIK3CA Status Mutated interacted with Treatment Other |  | 0.51 | 2.40 | 0.08 | 3.21 |
| Patients (mutated, wild)=3160 (1366, 1794) | Study arms=31, Pubs.=11 | AIC=55.05 | Q=63.33 | Q p-value=0.00 | I2=19.72% |
| *Note:* |  |  |  |  |  |
| Significance codes: 0.001 " *** " 0.01 " ** " 0.05 " * " |  |  |  |  |  |
| ^1^ Other treatment arm may include targeted therapy, standard maintenance chemotherapy or immunotherapy. |  |  |  |  |  |

AIC, Akaike Information Criterion; CI, confidence interval; ctDNA, circulating tumor deoxyribose nucleic acid; PIK3CA, phosphatidylinositol-4,5-bisphosphate 3-kinase catalytic subunit alpha; PFS, progression-free survival; Q, Test statistic for (residual) heterogeneity, Q p-value, p-value for test for (residual) heterogeneity, I^2^ , I^2^ statistic in %; SE, standard error

**Notes:**
[1] All estimates provided represent odds ratios surviving at 6 months.
[2] All models included random effects at the study and publication level.

## Table S6. Meta-regression results for 12-month PFS rates; models without interactions

Association of PIK3CA mutation with 12 month survival rate (odds ratio): Base model

| **Variable** | **Estimate** | **SE** | **CI-Lower** | **CI-Upper** | **P-value** |
| --- | --- | --- | --- | --- | --- |
| Intercept | 0.57 | 1.46 | 0.26 | 1.27 | P = 0.16 |
| PIK3CA Status Mutated | 0.76 | 1.13 | 0.59 | 0.99 | P < 0.05 * |
| Patients (mutated, wild)=2468 (1056, 1412) | Study arms=22, Pubs.=10 | AIC=71.95 | Q=215.87 | Q p-value=0.00 | I2=42.02% |
| *Note:* |  |  |  |  |  |
| Significance codes: 0.001 " *** " 0.01 " ** " 0.05 " * " |  |  |  |  |  |

Base model + controlling for ctDNA testing methodology

| **Variable** | **Estimate** | **SE** | **CI-Lower** | **CI-Upper** | **P-value** |
| --- | --- | --- | --- | --- | --- |
| Intercept | 0.42 | 1.39 | 0.21 | 0.85 | P < 0.05 * |
| PIK3CA Status Mutated | 0.77 | 1.15 | 0.58 | 1.03 | P = 0.08 |
| ctDNA Testing Tissue testing | 1.19 | 1.17 | 0.86 | 1.66 | P = 0.28 |
| Patients (mutated, wild)=2104 (952, 1152) | Study arms=20, Pubs.=9 | AIC=67.07 | Q=96.52 | Q p-value=0.00 | I2=39.77% |
| *Note:* |  |  |  |  |  |
| Significance codes: 0.001 " *** " 0.01 " ** " 0.05 " * " |  |  |  |  |  |

Base model + controlling for fulvestrant treatment

| **Variable** | **Estimate** | **SE** | **CI-Lower** | **CI-Upper** | **P-value** |
| --- | --- | --- | --- | --- | --- |
| Intercept | 1.13 | 1.99 | 0.27 | 4.75 | P = 0.86 |
| PIK3CA Status Mutated | 0.74 | 1.13 | 0.57 | 0.94 | P < 0.05 * |
| Fulvestrant Treatment Yes | 0.41 | 2.20 | 0.08 | 2.14 | P = 0.27 |
| Patients (mutated, wild)=2468 (1056, 1412) | Study arms=22, Pubs.=10 | AIC=72.94 | Q=208.53 | Q p-value=0.00 | I2=46.18% |
| *Note:* |  |  |  |  |  |
| Significance codes: 0.001 " *** " 0.01 " ** " 0.05 " * " |  |  |  |  |  |

Base model + controlling for study treatment

| **Variable** | **Estimate** | **SE** | **CI-Lower** | **CI-Upper** | **P-value** |
| --- | --- | --- | --- | --- | --- |
| Intercept | 0.99 | 1.52 | 0.41 | 2.38 | P = 0.97 |
| PIK3CA Status Mutated | 0.68 | 1.13 | 0.53 | 0.88 | P < 0.01 ** |
| Treatment Everolimus + Exemestane | 0.58 | 2.30 | 0.10 | 3.39 | P = 0.52 |
| Treatment Placebo + Exemestane | 0.13 | 2.44 | 0.02 | 0.85 | P < 0.05 * |
| Treatment Placebo + Fulvestrant | 0.45 | 1.34 | 0.24 | 0.83 | P < 0.05 * |
| Treatment Other | 3.23 | 2.32 | 0.54 | 19.26 | P = 0.18 |
| Patients (mutated, wild)=2468 (1056, 1412) | Study arms=22, Pubs.=10 | AIC=50.3 | Q=34.23 | Q p-value=0.01 | I2=48.46% |
| *Note:* |  |  |  |  |  |
| Significance codes: 0.001 " *** " 0.01 " ** " 0.05 " * " |  |  |  |  |  |
| ^1^ Other treatment arm may include targeted therapy, standard maintenance chemotherapy or immunotherapy. |  |  |  |  |  |

AIC, Akaike Information Criterion; CI, confidence interval; ctDNA, circulating tumor deoxyribose nucleic acid; PIK3CA, phosphatidylinositol-4,5-bisphosphate 3-kinase catalytic subunit alpha; PFS, progression-free survival; Q, Test statistic for (residual) heterogeneity, Q p-value, p-value for test for (residual) heterogeneity, I^2^ , I^2^ statistic in %; SE, standard error

**Notes:**
[1] All estimates provided represent odds ratios surviving at 12 months.
[2] All models included random effects at the study and publication level.

## Table S7. Meta-regression results for 12-month PFS rates; models with interactions

Base model + controlling for ctDNA testing and interaction with PIK3CA status

| **Variable** | **Estimate** | **SE** | **CI-Lower** | **CI-Upper** | **P-value** |
| --- | --- | --- | --- | --- | --- |
| Intercept | 0.42 | 1.39 | 0.21 | 0.86 | P < 0.05 * |
| PIK3CA Status Mutated | 0.76 | 1.17 | 0.54 | 1.07 | P = 0.11 |
| ctDNA Testing Tissue testing | 1.14 | 1.30 | 0.66 | 1.98 | P = 0.61 |
| PIK3CA Status Mutated interacted with ctDNA Testing Tissue testing | 1.06 | 1.39 | 0.53 | 2.13 | P = 0.85 |
| Patients (mutated, wild)=2104 (952, 1152) | Study arms=20, Pubs.=9 | AIC=69.29 | Q=96.34 | Q p-value=0.00 | I2=39.79% |
| *Note:* |  |  |  |  |  |
| Significance codes: 0.001 " *** " 0.01 " ** " 0.05 " * " |  |  |  |  |  |

[1] “PIk3CA status association among studies with tissue testing: -0.21 [-0.79,0.37]”

Base model + controlling for fulvestrant treatment and interaction with PIK3CA status

| **Variable** | **Estimate** | **SE** | **CI-Lower** | **CI-Upper** | **P-value** |
| --- | --- | --- | --- | --- | --- |
| Intercept | 1.24 | 2.03 | 0.28 | 5.47 | P = 0.77 |
| PIK3CA Status Mutated | 0.65 | 1.23 | 0.42 | 1.02 | P = 0.06 |
| Fulvestrant Treatment Yes | 0.34 | 2.30 | 0.06 | 1.95 | P = 0.21 |
| PIK3CA Status Mutated interacted with Fulvestrant Treatment Yes | 1.29 | 1.32 | 0.72 | 2.33 | P = 0.37 |
| Patients (mutated, wild)=2468 (1056, 1412) | Study arms=22, Pubs.=10 | AIC=74.63 | Q=207.54 | Q p-value=0.00 | I2=42.08% |
| *Note:* |  |  |  |  |  |
| Significance codes: 0.001 " *** " 0.01 " ** " 0.05 " * " |  |  |  |  |  |

Base model + controlling for study treatment and interaction with PIK3CA status

| **Variable** | **Estimate** | **SE** | **CI-Lower** | **CI-Upper** | **P-value** |
| --- | --- | --- | --- | --- | --- |
| Intercept | 0.70 | 1.75 | 0.21 | 2.34 | P = 0.53 |
| PIK3CA Status Mutated | 1.87 | 1.90 | 0.47 | 7.49 | P = 0.35 |
| Treatment Everolimus + Exemestane | 0.83 | 2.97 | 0.08 | 8.71 | P = 0.86 |
| Treatment Placebo + Exemestane | 0.17 | 3.10 | 0.02 | 2.01 | P = 0.15 |
| Treatment Placebo + Fulvestrant | 0.49 | 1.47 | 0.21 | 1.13 | P = 0.09 |
| Treatment Other | 4.64 | 2.99 | 0.44 | 49.50 | P = 0.18 |
| PIK3CA Status Mutated interacted with Treatment Everolimus + Exemestane | 0.32 | 3.09 | 0.03 | 3.62 | P = 0.33 |
| PIK3CA Status Mutated interacted with Treatment Placebo + Fulvestrant | 0.63 | 1.69 | 0.20 | 1.95 | P = 0.39 |
| PIK3CA Status Mutated interacted with Treatment Other | 0.33 | 3.16 | 0.03 | 3.98 | P = 0.35 |
| Patients (mutated, wild)=2468 (1056, 1412) | Study arms=22, Pubs.=10 | AIC=53.74 | Q=31.26 | Q p-value=0.00 | I2=4.36% |
| *Note:* |  |  |  |  |  |
| Significance codes: 0.001 " *** " 0.01 " ** " 0.05 " * " |  |  |  |  |  |
| ^1^ Other treatment arm may include targeted therapy, standard maintenance chemotherapy or immunotherapy. |  |  |  |  |  |

AIC, Akaike Information Criterion; CI, confidence interval; ctDNA, circulating tumor deoxyribose nucleic acid; PIK3CA, phosphatidylinositol-4,5-bisphosphate 3-kinase catalytic subunit alpha; PFS, progression-free survival; Q, Test statistic for (residual) heterogeneity, Q p-value, p-value for test for (residual) heterogeneity, I^2^ , I^2^ statistic in %; SE, standard error

**Notes:**
[1] All estimates provided represent odds ratios surviving at 12 months.
[2] All models included random effects at the study and publication level.

## Table S8. Meta-regression results for 18-month PFS rates; models without interactions

Association of PIK3CA mutation with 18 month survival rate (odds ratio): Base model

| **Variable** | **Estimate** | **SE** | **CI-Lower** | **CI-Upper** | **P-value** |
| --- | --- | --- | --- | --- | --- |
| Intercept | 0.49 | 1.52 | 0.20 | 1.24 | P = 0.12 |
| PIK3CA Status Mutated | 0.83 | 1.21 | 0.54 | 1.26 | P = 0.35 |
| Patients (mutated, wild)=1726 (811, 915) | Study arms=14, Pubs.=6 | AIC=41.23 | Q=110.18 | Q p-value=0.00 | I2=31.72% |
| *Note:* |  |  |  |  |  |
| Significance codes: 0.001 " *** " 0.01 " ** " 0.05 " * " |  |  |  |  |  |

Base model + controlling for ctDNA testing methodology

| **Variable** | **Estimate** | **SE** | **CI-Lower** | **CI-Upper** | **P-value** |
| --- | --- | --- | --- | --- | --- |
| Intercept | 0.40 | 1.40 | 0.19 | 0.85 | P < 0.05 * |
| PIK3CA Status Mutated | 0.86 | 1.23 | 0.54 | 1.38 | P = 0.49 |
| ctDNA Testing Tissue testing | 0.82 | 1.56 | 0.30 | 2.24 | P = 0.66 |
| Patients (mutated, wild)=1362 (707, 655) | Study arms=12, Pubs.=5 | AIC=38.94 | Q=36.28 | Q p-value=0.00 | I2=31.55% |
| *Note:* |  |  |  |  |  |
| Significance codes: 0.001 " *** " 0.01 " ** " 0.05 " * " |  |  |  |  |  |

Base model + controlling for fulvestrant treatment

| **Variable** | **Estimate** | **SE** | **CI-Lower** | **CI-Upper** | **P-value** |
| --- | --- | --- | --- | --- | --- |
| Intercept | 0.60 | 1.78 | 0.17 | 2.12 | P = 0.39 |
| PIK3CA Status Mutated | 0.82 | 1.21 | 0.54 | 1.24 | P = 0.31 |
| Fulvestrant Treatment Yes | 0.74 | 1.85 | 0.19 | 2.86 | P = 0.63 |
| Patients (mutated, wild)=1726 (811, 915) | Study arms=14, Pubs.=6 | AIC=43.14 | Q=99.96 | Q p-value=0.00 | I2=33.89% |
| *Note:* |  |  |  |  |  |
| Significance codes: 0.001 " *** " 0.01 " ** " 0.05 " * " |  |  |  |  |  |

Base model + controlling for study treatment

| **Variable** | **Estimate** | **SE** | **CI-Lower** | **CI-Upper** | **P-value** |
| --- | --- | --- | --- | --- | --- |
| Intercept | 1.05 | 1.22 | 0.67 | 1.65 | P = 0.81 |
| PIK3CA Status Mutated | 0.75 | 1.16 | 0.53 | 1.04 | P = 0.08 |
| Treatment Everolimus + Exemestane | 0.27 | 1.28 | 0.15 | 0.48 | P < 0.001 *** |
| Treatment Placebo + Exemestane | 0.03 | 7.08 | 0.00 | 2.90 | P = 0.12 |
| Treatment Placebo + Fulvestrant | 0.37 | 1.25 | 0.22 | 0.61 | P < 0.01 ** |
| Treatment Other | 1.62 | 1.26 | 0.95 | 2.74 | P = 0.07 |
| Patients (mutated, wild)=1726 (811, 915) | Study arms=14, Pubs.=6 | AIC=26.07 | Q=5.71 | Q p-value=0.68 | I2=0% |
| *Note:* |  |  |  |  |  |
| Significance codes: 0.001 " *** " 0.01 " ** " 0.05 " * " |  |  |  |  |  |
| ^1^ Other treatment arm may include targeted therapy, standard maintenance chemotherapy or immunotherapy. |  |  |  |  |  |

AIC, Akaike Information Criterion; CI, confidence interval; ctDNA, circulating tumor deoxyribose nucleic acid; PIK3CA, phosphatidylinositol-4,5-bisphosphate 3-kinase catalytic subunit alpha; PFS, progression-free survival; Q, Test statistic for (residual) heterogeneity, Q p-value, p-value for test for (residual) heterogeneity, I^2^ , I^2^ statistic in %; SE, standard error

**Notes:**
[1] All estimates provided represent odds ratios surviving at 18 months.
[2] All models included random effects at the study and publication level.

## Table S9. Meta-regression results for 18-month PFS rates; models with interactions

Base model + controlling for ctDNA testing and interaction with PIK3CA status

| **Variable** | **Estimate** | **SE** | **CI-Lower** | **CI-Upper** | **P-value** |
| --- | --- | --- | --- | --- | --- |
| Intercept | 0.40 | 1.39 | 0.19 | 0.86 | P < 0.05 * |
| PIK3CA Status Mutated | 0.85 | 1.23 | 0.53 | 1.38 | P = 0.47 |
| ctDNA Testing Tissue testing | 0.32 | 9.98 | 0.00 | 64.30 | P = 0.63 |
| PIK3CA Status Mutated interacted with ctDNA Testing Tissue testing | 2.58 | 9.66 | 0.01 | 481.08 | P = 0.69 |
| Patients (mutated, wild)=1362 (707, 655) | Study arms=12, Pubs.=5 | AIC=40.75 | Q=35.94 | Q p-value=0.00 | I2=32.2% |
| *Note:* |  |  |  |  |  |
| Significance codes: 0.001 " *** " 0.01 " ** " 0.05 " * " |  |  |  |  |  |

Base model + controlling for fulvestrant treatment and interaction with PIK3CA status

| **Variable** | **Estimate** | **SE** | **CI-Lower** | **CI-Upper** | **P-value** |
| --- | --- | --- | --- | --- | --- |
| Intercept | 0.66 | 2.06 | 0.13 | 3.30 | P = 0.58 |
| PIK3CA Status Mutated | 0.78 | 1.35 | 0.40 | 1.51 | P = 0.42 |
| Fulvestrant Treatment Yes | 0.60 | 2.58 | 0.07 | 5.00 | P = 0.61 |
| PIK3CA Status Mutated interacted with Fulvestrant Treatment Yes | 1.13 | 1.51 | 0.45 | 2.83 | P = 0.77 |
| Patients (mutated, wild)=1726 (811, 915) | Study arms=14, Pubs.=6 | AIC=45.19 | Q=99.61 | Q p-value=0.00 | I2=31.32% |
| *Note:* |  |  |  |  |  |
| Significance codes: 0.001 " *** " 0.01 " ** " 0.05 " * " |  |  |  |  |  |

Base model + controlling for study treatment and interaction with PIK3CA status

| **Variable** | **Estimate** | **SE** | **CI-Lower** | **CI-Upper** | **P-value** |
| --- | --- | --- | --- | --- | --- |
| Intercept | 0.95 | 1.30 | 0.49 | 1.87 | P = 0.86 |
| PIK3CA Status Mutated | 0.92 | 1.51 | 0.32 | 2.65 | P = 0.86 |
| Treatment Everolimus + Exemestane | 0.25 | 1.44 | 0.10 | 0.64 | P < 0.05 * |
| Treatment Placebo + Exemestane | 0.04 | 7.15 | 0.00 | 5.50 | P = 0.15 |
| Treatment Placebo + Fulvestrant | 0.43 | 1.38 | 0.19 | 0.99 | P < 0.05 * |
| Treatment Other | 1.94 | 1.38 | 0.85 | 4.43 | P = 0.09 |
| PIK3CA Status Mutated interacted with Treatment Everolimus + Exemestane | 1.15 | 1.72 | 0.28 | 4.61 | P = 0.81 |
| PIK3CA Status Mutated interacted with Treatment Placebo + Fulvestrant | 0.73 | 1.61 | 0.22 | 2.46 | P = 0.53 |
| PIK3CA Status Mutated interacted with Treatment Other | 0.62 | 1.68 | 0.16 | 2.33 | P = 0.39 |
| Patients (mutated, wild)=1726 (811, 915) | Study arms=14, Pubs.=6 | AIC=30.95 | Q=3.29 | Q p-value=0.66 | I2=6.43% |
| *Note:* |  |  |  |  |  |
| Significance codes: 0.001 " *** " 0.01 " ** " 0.05 " * " |  |  |  |  |  |
| ^1^ Other treatment arm may include targeted therapy, standard maintenance chemotherapy or immunotherapy. |  |  |  |  |  |

AIC, Akaike Information Criterion; CI, confidence interval; ctDNA, circulating tumor deoxyribose nucleic acid; PIK3CA, phosphatidylinositol-4,5-bisphosphate 3-kinase catalytic subunit alpha; PFS, progression-free survival; Q, Test statistic for (residual) heterogeneity, Q p-value, p-value for test for (residual) heterogeneity, I^2^ , I^2^ statistic in %; SE, standard error

**Notes:**
[1] All estimates provided represent odds ratios surviving at 18 months.
[2] All models included random effects at the study and publication level.

## Figure S1. Within-study differences between PIK3CA-mutated and wild-type cohorts (a) 12-month PFS rate (odds ratio) (b) 18-month PFS rate (odds ratio)


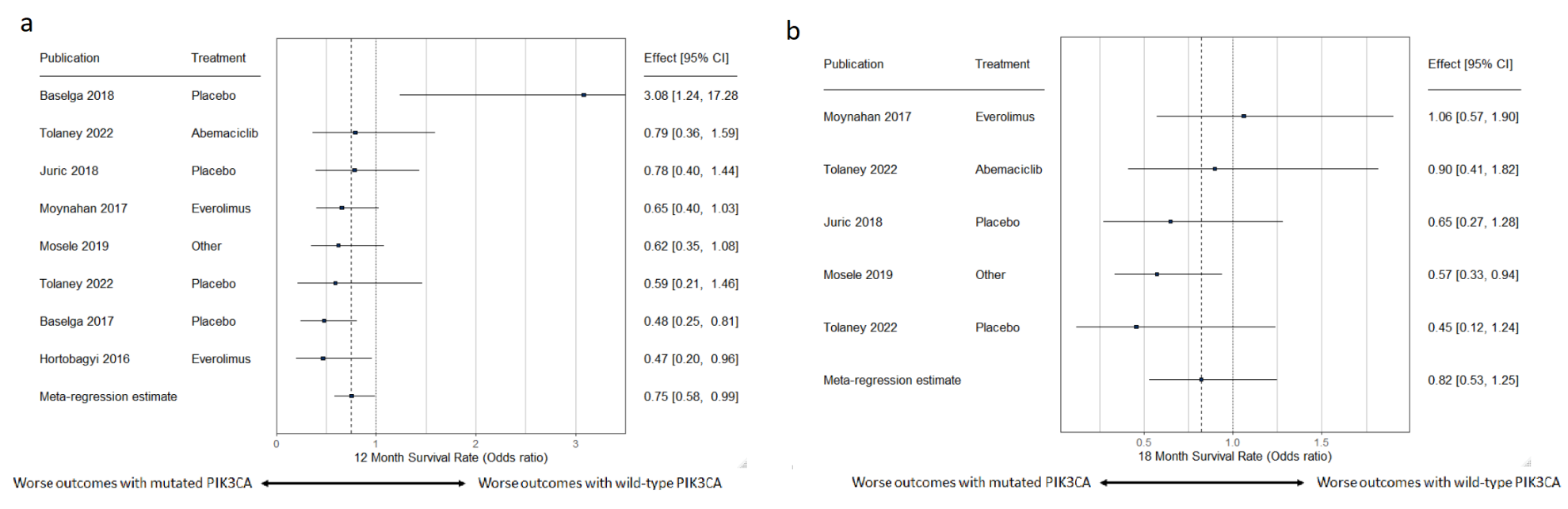


CI, confidence interval; PIK3CA, phosphatidylinositol-4,5-bisphosphate 3-kinase catalytic subunit alpha; PFS, progression-free survival

# References

1. Mollon LE, Anderson EJ, Dean JL, et al. A Systematic Literature Review of the Prognostic and Predictive Value of PIK3CA Mutations in HR+/HER2− Metastatic Breast Cancer. *Clinical breast cancer.* 2020;20(3):e232-e243.

2. Guyot P, Ades AE, Ouwens MJ, Welton NJ. Enhanced secondary analysis of survival data: reconstructing the data from published Kaplan-Meier survival curves. *BMC Med Res Methodol.* 2012;12:9.

3. Latimer NR. Survival Analysis for Economic Evaluations Alongside Clinical Trials—Extrapolation with Patient-Level Data:Inconsistencies, Limitations, and a Practical Guide. *Medical Decision Making.* 2013;33(6):743-754.

4. Schemper M, Smith TL. A note on quantifying follow-up in studies of failure time. *Control Clin Trials.* 1996;17(4):343-346.

5. Andre F, Ciruelos EM, Juric D, et al. Alpelisib plus fulvestrant for PIK3CA-mutated, hormone receptor-positive, human epidermal growth factor receptor-2-negative advanced breast cancer: final overall survival results from SOLAR-1. *Ann Oncol.* 2021;32(2):208-217.

6. Juric D, Loibl S, Andre F, et al. Alpelisib (ALP) with fulvestrant (FUL) in patients (pts) with PIK3CA-mutated hormone receptor-positive (HR+), human epidermal growth factor receptor-2-negative (HER2-) advanced breast cancer (ABC): Primary or secondary resistance to prior endocrine therapy (ET) in the SOLAR-1 trial. *Clin Oncol.* 2019;37(Suppl 15):1038-1038.

7. Baselga J, Im SA, Iwata H, et al. Buparlisib plus fulvestrant versus placebo plus fulvestrant in postmenopausal, hormone receptor-positive, HER2-negative, advanced breast cancer (BELLE-2): a randomised, double-blind, placebo-controlled, phase 3 trial. *Lancet Oncol.* 2017;18(7):904-916.

8. Campone M, Im SA, Iwata H, et al. Buparlisib plus fulvestrant versus placebo plus fulvestrant for postmenopausal, hormone receptor-positive, human epidermal growth factor receptor 2-negative, advanced breast cancer: Overall survival results from BELLE-2. *Eur J Cancer.* 2018;103:147-154.

9. Cristofanilli M, Turner NC, Bondarenko I, et al. Fulvestrant plus palbociclib versus fulvestrant plus placebo for treatment of hormone-receptor-positive, HER2-negative metastatic breast cancer that progressed on previous endocrine therapy (PALOMA-3): final analysis of the multicentre, double-blind, phase 3 randomised controlled trial. *Lancet Oncol.* 2016;17(4):425-439.

10. Turner NC, Slamon DJ, Ro J, et al. Overall Survival with Palbociclib and Fulvestrant in Advanced Breast Cancer. *N Engl J Med.* 2018;379(20):1926-1936.

11. Di Leo A, Johnston S, Lee KS, et al. Buparlisib plus fulvestrant in postmenopausal women with hormone-receptor-positive, HER2-negative, advanced breast cancer progressing on or after mTOR inhibition (BELLE-3): a randomised, double-blind, placebo-controlled, phase 3 trial. *Lancet Oncol.* 2018;19(1):87-100.

12. Dickler MN, Saura C, Richards DA, et al. Phase II Study of Taselisib (GDC-0032) in Combination with Fulvestrant in Patients with HER2-Negative, Hormone Receptor-Positive Advanced Breast Cancer. *Clin Cancer Res.* 2018;24(18):4380-4387.

13. Fleming GF, Ma CX, Huo D, et al. Phase II trial of temsirolimus in patients with metastatic breast cancer. *Breast Cancer Res Treat.* 2012;136(2):355-363.

14. Hortobagyi GN, Chen D, Piccart M, et al. Correlative Analysis of Genetic Alterations and Everolimus Benefit in Hormone Receptor-Positive, Human Epidermal Growth Factor Receptor 2-Negative Advanced Breast Cancer: Results From BOLERO-2. *J Clin Oncol.* 2016;34(5):419-426.

15. Krop IE, Mayer IA, Ganju V, et al. Pictilisib for oestrogen receptor-positive, aromatase inhibitor-resistant, advanced or metastatic breast cancer (FERGI): a randomised, double-blind, placebo-controlled, phase 2 trial. *Lancet Oncol.* 2016;17(6):811-821.

16. Mayer IA, Abramson VG, Formisano L, et al. A Phase Ib Study of Alpelisib (BYL719), a PI3Kalpha-Specific Inhibitor, with Letrozole in ER+/HER2- Metastatic Breast Cancer. *Clin Cancer Res.* 2017;23(1):26-34.

17. Moynahan ME, Chen D, He W, et al. Correlation between PIK3CA mutations in cell-free DNA and everolimus efficacy in HR(+), HER2(-) advanced breast cancer: results from BOLERO-2. *Br J Cancer.* 2017;116(6):726-730.

18. Mosele F, Verret B, Lusque A, et al. Abstract 4895: Natural history and outcome of patients presenting a metastatic breast cancer with PIK3CA mutation. *Cancer Res.* 2019;79(Suppl 13 ):4895.

19. Baselga J, Dent SF, Cortes J, et al. Phase III study of taselisib (GDC-0032) + fulvestrant (FULV) v FULV in patients (pts) with estrogen receptor (ER)-positive, PIK3CA-mutant (MUT), locally advanced or metastatic breast cancer (MBC): Primary analysis from SANDPIPER. *Clin Oncol.* 2018;36(18).

20. Tolaney SM, Toi M, Neven P, et al. Abstract 4458: Clinical significance of PIK3CA and ESR1 mutations in ctDNA and FFPE samples from the MONARCH 2 study of abemaciclib plus fulvestrant. *Cancer Research.* 2019;79(Suppl 13 ):4458.
